# Mouse nuclear RNAi-defective 2 promotes splicing of weak 5′ splice sites

**DOI:** 10.1261/rna.079465.122

**Published:** 2023-08

**Authors:** Matyas Flemr, Michaela Schwaiger, Daniel Hess, Vytautas Iesmantavicius, Josip Ahel, Alex Charles Tuck, Fabio Mohn, Marc Bühler

**Affiliations:** 1 Friedrich Miescher Institute for Biomedical Research, 4058 Basel, Switzerland; 2 Swiss Institute of Bioinformatics, 4058 Basel, Switzerland; 3 University of Basel, 4003 Basel, Switzerland

**Keywords:** NRDE2, CCDC174, CLIP, RNA binding, U1 snRNP, weak 5′ splice site, splice site selection

## Abstract

Removal of introns during pre-mRNA splicing, which is central to gene expression, initiates by base pairing of U1 snRNA with a 5′ splice site (5′SS). In mammals, many introns contain weak 5′SSs that are not efficiently recognized by the canonical U1 snRNP, suggesting alternative mechanisms exist. Here, we develop a cross-linking immunoprecipitation coupled to a high-throughput sequencing method, BCLIP-seq, to identify NRDE2 (nuclear RNAi-defective 2), and CCDC174 (coiled-coil domain-containing 174) as novel RNA-binding proteins in mouse ES cells that associate with U1 snRNA and 5′SSs. Both proteins bind directly to U1 snRNA independently of canonical U1 snRNP-specific proteins, and they are required for the selection and effective processing of weak 5′SSs. Our results reveal that mammalian cells use noncanonical splicing factors bound directly to U1 snRNA to effectively select suboptimal 5′SS sequences in hundreds of genes, promoting proper splice site choice, and accurate pre-mRNA splicing.

## INTRODUCTION

Accurate removal of noncoding intronic sequences during pre-mRNA splicing is a prerequisite for eukaryotic gene expression. Most introns are excised by the major spliceosome, a dynamic macromolecular assembly consisting of five small nuclear ribonucleoprotein particles (U1, U2, U4, U5, and U6 snRNPs) and a myriad of associated splicing factors (Wahl et al. 2009). The highly structured snRNA components of snRNPs bind complementary sequences in pre-mRNA that define and position the splice sites to form a catalytic spliceosome, in which two consecutive transesterification reactions liberate the intron and allow two neighboring exons to be ligated (Shi 2017; Kastner et al. 2019; Wilkinson et al. 2019).

5′ splice site (5′SS) recognition by the U1 snRNP represents the first step of spliceosome assembly. 5′SSs comprise a short motif that is recognized by a complementary sequence at the 5′ end of the U1 snRNA (Kondo et al. 2015; Plaschka et al. 2018). However, major differences exist between model organisms in which pre-mRNA splicing has been investigated. In budding yeast, an almost invariant 5′SS motif is bound by a U1 snRNP that triggers spliceosome assembly and rapid cotranscriptional splicing shortly after the intron has been transcribed (Lacadie and Rosbash 2005; Carrillo Oesterreich et al. 2016). In contrast, in other eukaryotes, ranging from fission yeast to mammals, U1 snRNPs must cope with a highly degenerate 5′SS motif (Fair and Pleiss 2017). The sequence variability of 5′SSs can decrease U1 binding affinity, resulting in weak 5′SSs with reduced splicing efficiency (Roca et al. 2013). Most mammalian genes contain multiple introns with several potential splice sites, among which the weak 5′SSs are often associated with alternative splicing patterns (Boutz et al. 2015; Lee and Rio 2015; Drexler et al. 2020). These include alternative exon inclusion, exon skipping and intron retention, which can all possess regulatory functions by generating different protein isoforms or ensuring the timely expression of mature transcripts (Mauger et al. 2016; Naro et al. 2017; Ule and Blencowe 2019). However, alternative processing of weak 5′SSs can also lead to nonfunctional transcripts targeted for degradation by RNA surveillance pathways (Davidson et al. 2012; Bresson et al. 2015; Peck et al. 2019). Proper choice and efficient splicing of weak 5′SSs are therefore crucial for accurate gene expression; that is, mutations decreasing splice site strength or impairing activity of splicing factors are a frequent cause of human genetic disorders (Wickramasinghe et al. 2015; Scotti and Swanson 2016; Anna and Monika 2018).

Due to the limited complementarity to U1 snRNA, weak 5′SSs are not efficiently recognized by the canonical U1 snRNP consisting of the U1 snRNA, a set of seven Sm proteins forming a ring structure common to other U snRNPs, and three U1-specific proteins, U1A, U1C, and U1-70K (Kondo et al. 2015). Therefore, additional splicing factors must be involved in efficient U1 binding and processing of weak 5′SSs. Despite a plethora of known splicing factors, additional proteins with uncharacterized splicing-related functions continue to be discovered. For example, NRDE2 (nuclear RNAi-defective 2) is a conserved protein with homologs detected in eukaryotes ranging from fission yeast to human, but absent in budding yeast. The fission yeast homolog Nrl1 has been shown to form a complex with Mtl1 (MTREX in mammals) and Ctr1 proteins (CCDC174 in mammals, for coiled-coil domain-containing 174) and interact with core splicing factors. The Nrl1–Ctr1–Mtl1 complex has been proposed to regulate the splicing of cryptic introns and target unspliced transcripts for degradation by the nuclear exosome (Lee et al. 2013; Zhou et al. 2015). In contrast, mammalian NRDE2 has been suggested to reduce exosome activity by inhibiting the MTREX helicase (Wang et al. 2019). A NRDE2 interaction with CCDC174 has thus far not been detected (Richard et al. 2018). Nevertheless, human CCDC174 was shown to interact with a splicing-related exon junction complex (EJC) component EIF4A3 (Volodarsky et al. 2015). NRDE2 was also found to interact with splicing factors, and its depletion led to the retention of weakly spliced introns (Jiao et al. 2019). However, potential mechanisms by which NRDE2 and CCDC174 might contribute to pre-mRNA splicing remain unknown, partly due to a lack of any predicted functional domains in these two largely uncharacterized proteins.

Here, we combined genome engineering in mouse embryonic stem cells (mESCs) with proteomics and genomics approaches to analyze the function of NRDE2 in mammalian cells. In order to reliably detect potential RNA substrates of NRDE2 with high sensitivity and at a transcriptome-wide level, we have developed a modified cross-linking immunoprecipitation coupled to next-generation sequencing method. This revealed that mouse NRDE2 together with CCDC174 bind to U1 snRNAs, which target NRDE2 to 5′SSs. At weak 5′SSs with suboptimal U1 complementarity, NRDE2 and CCDC174 are necessary for correct splice site choice and efficient pre-mRNA splicing. Thus, we reveal that mammalian cells use an alternative strategy involving direct U1 snRNA interaction with noncanonical splicing factors to select and splice introns with suboptimal 5′SSs.

## RESULTS

### NRDE2 is required for cell growth independently of its protein interactors

Using our previously established mESC line expressing 4-hydroxytamoxifen (4OHT) inducible Cre recombinase and bacterial BirA ligase (Flemr and Bühler 2015), we generated inducible *Nrde2* knockout cells. These cells were further edited to introduce homozygous *Nrde2* mutations and to tag NRDE2 with a fluorescent protein for live-cell imaging or with a composite 3xFLAG-AviTag (3A tag) for tandem FLAG-Streptavidin purifications. The knockout and tagging approaches were used on other endogenous genes in this study (Supplemental Fig. S1A; Supplemental Table S1), which also included fusions with the 2xHA-FKBP12^F36V^ domain (dTAG) for rapid protein depletion by the dTAG-13 compound (Nabet et al. 2018). We assessed the potential consequences of the tagging approach on protein function by comparing RNA-seq gene expression profiles with that of untagged cells, which revealed minor effects only in the case of 3A-tagging an EJC component EIF4A3 or dTAG tagging of CCDC174 and the snRNP component SmE (Supplemental Fig. S1B). The efficacy of 4OHT or dTAG-13-mediated protein depletion was verified by western blotting (Supplemental Fig. S1C,D).

**FIGURE 1.**
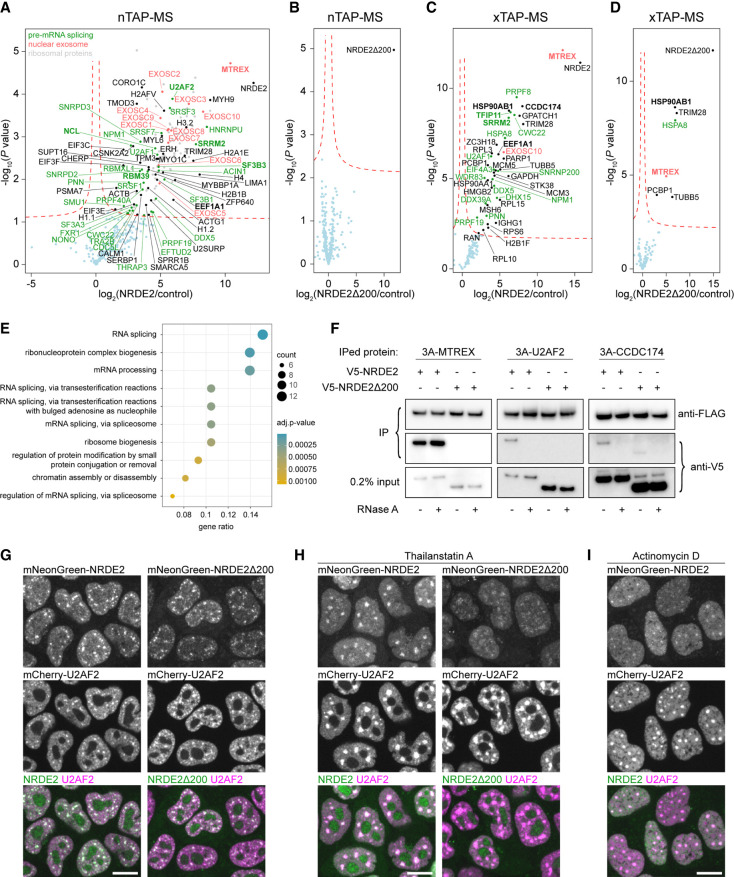
Protein interaction analysis links NRDE2 to pre-mRNA splicing. (*A*,*B*) Native TAP-MS analysis of 3A-NRDE2 (*A*) and 3A-NRDE2Δ200 (*B*) under low-salt conditions (100 mM NaCl) compared with untagged control and performed in three independent replicates for each sample. The red dashed line marks false discovery rate of 0.05. The components of the nuclear exosome are labeled in red. Proteins with GO term “RNA splicing” are depicted in green. Proteins highlighted in bold have also been identified in the yeast two-hybrid (Y2H) screen. Unlabeled gray dots represent significantly enriched ribosomal proteins. (*C*,*D*) Formaldehyde cross-linking TAP-MS analysis of 3A-NRDE2 (*C*) and 3A-NRDE2Δ200 (*D*) compared with untagged control and performed in three independent replicates for each sample. Same labeling scheme as in *A* and *B* applies. (*E*) GO term analysis of 87 high-confidence hits from a Y2H screen of full-length NRDE2 (see also Supplemental Table S2). (*F*) Streptavidin pull-down and coimmunoprecipitation of endogenous 3A-tagged proteins from cells transiently overexpressing V5-NRDE2 or V5-NRDE2Δ200 fused to 2A-Puro in the presence or absence of RNase A. (*G*–*I*) Live-cell spinning-disk microscopy of endogenous mNeonGreen-tagged NRDE2 and NRDE2Δ200 with endogenous mCherry-tagged NS marker U2AF2 (Chusainow et al. 2005). The imaging was performed in untreated cells (*G*), cells treated for 6 h with 1 µM splicing inhibitor Thailanstatin A (*H*), or cells treated for 2 h with 5 µg/mL transcription inhibitor Actinomycin D (*I*). Scale bar 10 µm.

In NRDE2 loss-of-function experiments, the 4OHT-induced knockout of *Nrde2* resulted in growth arrest (Supplemental Fig. S2A), revealing NRDE2 has an essential function in cellular growth. To understand the basis for this phenotype, we mapped the NRDE2 protein–protein interaction (PPI) network by performing tandem affinity purifications of 3A-NRDE2 under mild native conditions coupled with mass spectrometry (nTAP-MS). The NRDE2 interactome was dominated by ribosomal proteins, splicing factors, and included the complete nuclear exosome (Fig. 1A). The nuclear exosome-associated RNA helicase MTREX (formerly MTR4) was the highest-scoring interactor, consistent with published data (Wang et al. 2019). Reciprocal coimmunoprecipitation (co-IP) of 3A-MTREX confirmed this interaction, and NRDE2 truncation experiments revealed MTREX interacts with NRDE2 within a region including amino acids 101–200 (Supplemental Fig. S2B). Four conserved aspartates within this region (Supplemental Fig. S2C) resembled an MTREX Arch interaction motif (Thoms et al. 2015), and a single D174R point mutation in NRDE2 substantially reduced the interaction with MTREX (Supplemental Fig. S2D). NRDE2-D174R protein levels were lower compared with wild-type NRDE2 (Supplemental Fig. S2E), suggesting that MTREX controls NRDE2 stability. This was further supported by reduced NRDE2 levels upon *Mtrex* knockout (Supplemental Fig. S2F), which is in line with previously published data from human cells (Wang et al. 2019). However, the amino-terminal 200 amino acid truncation (NRDE2Δ200) was expressed at a level comparable with wild-type NRDE2. Thus, the amino-terminal region is required for NRDE2 destabilization in the absence of MTREX. MTREX and nuclear exosome components were lost from the NRDE2-D174R nTAP-MS PPI network, whereas ribosomal proteins and several splicing factors were still present (Supplemental Fig. S2G). Strikingly, we did not detect any PPIs for NRDE2Δ200 under these conditions (Fig. 1B), indicating the amino-terminal region of NRDE2 is essential for interactions with other proteins. The expression of NRDE2-D174R or NRDE2Δ200 promoted cell viability, albeit at a slower growth rate in the latter case, with cell growth being arrested upon induced knockout of the *Nrde2-D174R* and *Nrde2*Δ*200* genes (Supplemental Fig. S2A). Thus, NRDE2 sustains cell growth independently of its protein interactors.

### NRDE2 localization to nuclear speckles depends on active pre-mRNA splicing

NRDE2 has a relatively large PPI network. To identify proteins in direct proximity to NRDE2 in vivo, we developed a limited cross-linking TAP-MS (xTAP-MS) protocol, which uses a short fixation with a low-concentration formaldehyde followed by stringent lysis and washing conditions. NRDE2 xTAP-MS binding proteins were enriched for MTREX and splicing factors (Fig. 1C), including proteins involved in different splicing steps, such as the DDX5 helicase, which regulates U1 snRNP–5′SS interactions (Liu 2002), the U2AF1 component of the prespliceosome 3′ splice site (3′SS)-defining U2AF complex (Chen et al. 2017), the core spliceosome scaffold PRPF8 with 5′ exon-stabilizing proteins SRRM2/SRM300 and CWC22, which are present through all stages of active spliceosome rearrangements (Zhang et al. 2018, 2019), as well as late spliceosome disassembly factors DHX15/PRP43 and TFIP11 (Yoshimoto et al. 2009). In addition, CCDC174 was also detected by xTAP-MS, indicating that it comes in proximity to NRDE2 in mammalian cells. NRDE2-D174R cross-linked to a similar set of proteins, including MTREX (Supplemental Fig. S2H), suggesting that NRDE2-D174R and MTREX still colocalize in vivo despite their reduced binding affinity. In agreement with the native pull-down, only a few mostly high-abundant proteins were detected in NRDE2Δ200 xTAP-MS along with low levels of MTREX, but with no spliceosome components present (Fig. 1D).

The prevalence of splicing factors in the NRDE2 interactome was further confirmed by a Y2H screen (Fig. 1E), identifying MTREX and CCDC174 as high-confidence interactors together with factors from different stages of spliceosome assembly, including U2AF2 and TFIP11 (Supplemental Table S2). Selected Y2H interactions were validated by reciprocal co-IP. Unlike MTREX, NRDE2 interactions with U2AF2 and CCDC174 required an intact RNA component (Fig. 1F), and a weak RNase-sensitive interaction was also maintained between CCDC174 and NRDE2Δ200.

Consistent with a splicing-related activity, fluorescent light microscopy revealed that NRDE2 was strongly enriched in nuclear splicing speckles (NSs) (Fig. 1G). NSs are interchromatin granules with a high concentration of splicing factors that have been linked to post-transcriptional processing of retained introns harboring weak splice sites (Vargas et al. 2011; Girard et al. 2012; Galganski et al. 2017; Gordon et al. 2021). NRDE2Δ200 showed a very similar subcellular distribution to wild-type NRDE2, despite its lack of interactions with splicing factors (Fig. 1G). Chemical inhibition of splicing with Thailanstatin A (Liu et al. 2013) resulted in the NRDE2Δ200 signal becoming more dispersed and NRDE2-D174R accumulated in nucleoli, with wild-type NRDE2 remaining concentrated in enlarged NSs (Fig. 1H; Supplemental Fig. S3A,B). In contrast, a transcriptional shut-off by Actinomycin D led to a substantial reduction of NS signal even for wild-type NRDE2 (Fig. 1I; Supplemental Fig. S3C). Overall, these results suggest that NRDE2 is actively recruited to and associated with pre-mRNAs that undergo splicing, independently of its interaction with splicing factors.

### NRDE2 and CCDC174 bind to 5′ splice sites

**FIGURE 2.**
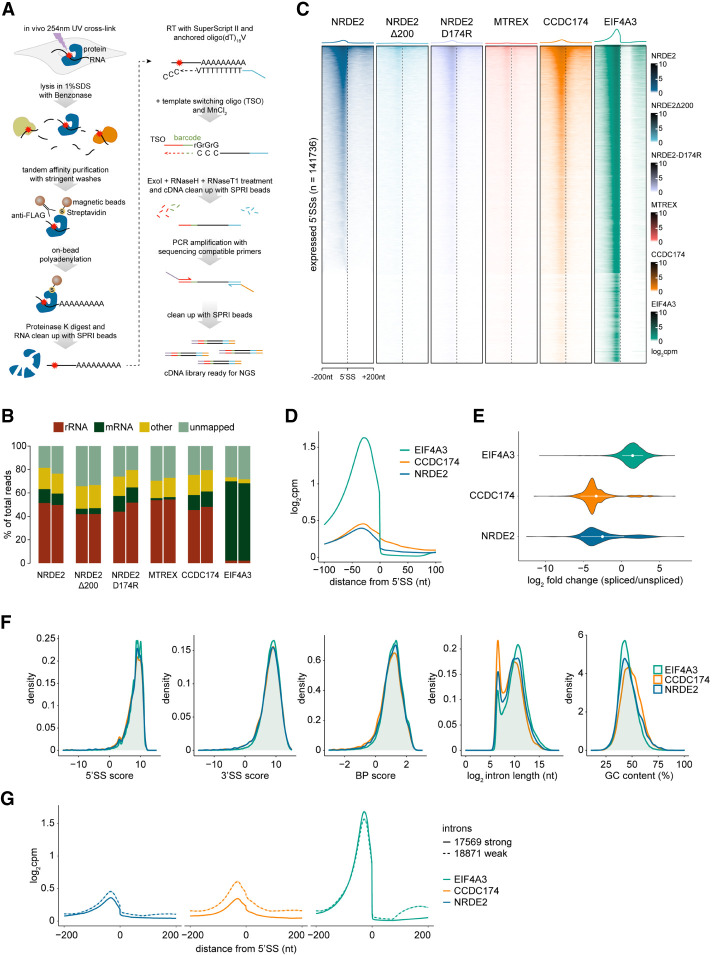
NRDE2 and CCDC174 bind to pre-mRNA 5′SSs. (*A*) Schematic workflow of the BCLIP-seq protocol. Template-switching oligo (TSO). (*B*) Mapping characteristics of the BCLIP-seq reads. The category “rRNA” contains reads mapping to mouse rDNA (GenBank: BK000964), category “mRNA” comprises reads mapping to protein-coding mature mRNAs and category “other” includes all remaining reads mapping to the mouse genome. The two bars in each sample represent two independent BCLIP-seq replicates. (*C*) Heat maps of the normalized BCLIP-seq signal intensity centered at annotated 5′SSs of all transcripts expressed in mESCs. Splice sites are ordered by decreasing NRDE2 signal. (*D*) Metaplots of normalized NRDE2, CCDC174, and EIF4A3 BCLIP-seq signal around all annotated expressed 5′SSs. (*E*) Violin plot showing ratios of spliced versus unspliced NRDE2, CCDC174, and EIF4A3 BCLIP-seq reads spanning annotated 5′SSs. Only 5′SSs of introns longer than 1 kb with a minimum of 10 splice site-overlapping reads were included in the analysis. White dots and bars represent the mean and standard deviation of all analyzed introns. (*F*) Density plots of the distribution of NRDE2, CCDC174, and EIF4A3 BCLIP-seq signal on all expressed introns ranked by their 5′SS strength, 3′SS strength, branch point strength, intron length, and GC content. (*G*) Metaplots of normalized NRDE2, CCDC174, and EIF4A3 BCLIP-seq signal around 5′SSs of introns selected based on their 5′SS, 3′SS, and branch point strength. Solid and dashed lines represent signal on introns with all three features above and below median strength, respectively.

To explore the RNA-binding potential of NRDE2, we developed a Benzonase-assisted RNA cross-linking immunoprecipitation protocol coupled to high-throughput sequencing (BCLIP-seq) (Fig. 2A; Lee and Ule 2018). Our approach offers a streamlined and sensitive alternative to existing CLIP techniques (Lee and Ule 2018), enabling us to perform RNA interaction profiling for low-abundant proteins such as NRDE2. The detergent-resistant Benzonase nuclease allows whole-cell lysis after UV cross-linking under highly denaturing conditions with simultaneous fragmentation of nucleic acids. The released protein–RNA cross-links are tandem affinity purified under stringent conditions, polyadenylated on beads, and the RNA is isolated for adaptor addition by a template switching-compatible reverse transcriptase (Turchinovich et al. 2014). This ligation-free cDNA library construction yields libraries of 20- to 150-bp-long inserts (Supplemental Fig. S4A).

We performed two BCLIP-seq replicates each for NRDE2, NRDE2Δ200, NRDE2-D174R, MTREX, and CCDC174. The EJC component EIF4A3 (Boehm and Gehring 2016) acted as a positive control for a splicing-dependent RNA-binding protein. The replicate samples correlated strongly (Supplemental Fig. S4B), which allowed us to merge the replicates into one data set for most of the subsequent analyses. More than half of the mapping reads in all variant NRDE2, MTREX, and CCDC174 libraries matched rRNA (Fig. 2B). MTREX was enriched at its known rRNA binding sites (Thoms et al. 2015), whereas no specific binding sites could be found for the NRDE2 variants, and CCDC174 was only mildly enriched in the 5′ external transcribed spacer (Supplemental Fig. S4C). The rRNA signal in NRDE2 and CCDC174 BCLIP-seq data could therefore represent nonspecific binding to abundant RNA. However, the EIF4A3 data indicated that rRNA was not a common contaminant of the BCLIP method (Fig. 2B). Therefore, we did not rule out potential functions of NRDE2 and CCDC174 in conjunction with rRNA.

Consistent with a potential role in pre-mRNA splicing, the majority of NRDE2 BCLIP peaks within protein-coding genes overlapped with exons and 5′SSs (Supplemental Fig. S4D). When considering all expressed 5′SSs, NRDE2 and, to a lesser extent, NRDE2-D174R were enriched at a subset of 5′SSs similar to CCDC174, whereas NRDE2Δ200 and MTREX showed very little binding around 5′SSs. EIF4A3 was enriched at most 5′SSs, as expected for an EJC factor (Fig. 2C). NRDE2 and CCDC174 signals peaked 30 nt upstream of 5′SSs, at the same position as EIF4A3, but, unlike EIF4A3, extended into introns. This was particularly prominent for CCDC174 (Fig. 2D). Both NRDE2 and CCDC174 bound mostly to unspliced 5′SSs, implying that they associate with pre-mRNA before or during splicing (Fig. 2E).

NRDE2- and CCDC174-bound 5′SSs showed a slight enrichment for shorter GC-rich introns; however, we could not distinguish them from control EIF4A3-bound 5′SSs by looking at the strength of individual intron-defining features, such as 5′SS, 3′SS, or branch point (Fig. 2F), which were calculated using the Matt toolkit (Gohr and Irimia 2018). Nevertheless, the overall NRDE2 and CCDC174 BCLIP signal was increased on 5′SSs of introns below median strength for all three features combined (Fig. 2G). Thus, NRDE2 and CCDC174 are RNA-binding proteins that associate with unspliced 5′SSs.

### *Nrde2* and *Mtrex* knockouts induce a 2C-like state

**FIGURE 3.**
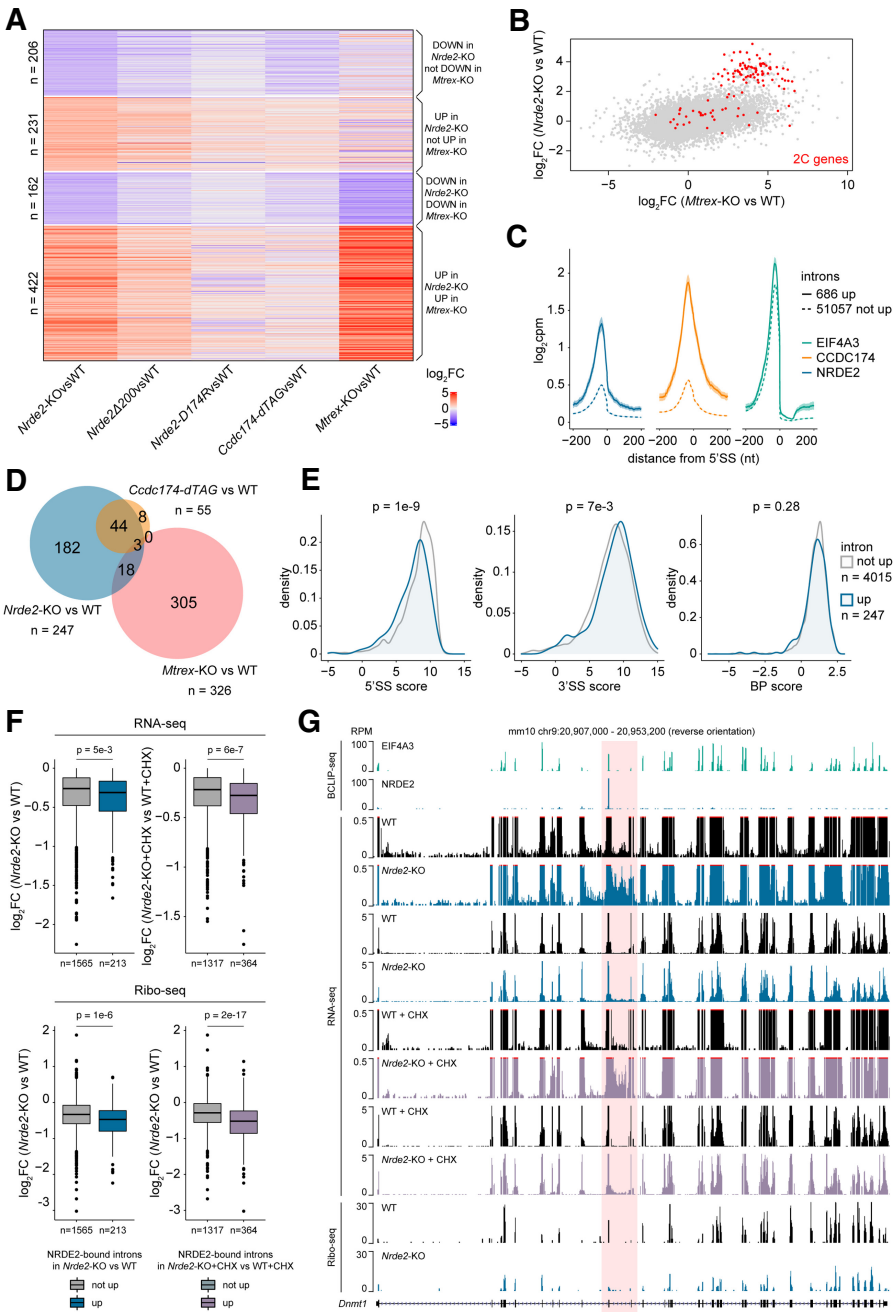
NRDE2 regulates the splicing of introns with weak 5′SSs. (*A*) Differential gene expression based on duplicate RNA-seq analysis of *Nrde2*-KO (4 d of 0.1 µM 4OHT treatment), *Nrde2*Δ*200*, *Nrde2-D174R*, *Ccdc174-dTAG* (24 h of 0.5 µM dTAG-13 treatment), and *Mtrex*-KO (3 d of 0.1 µM 4OHT treatment) cells compared with the matching untreated and wild-type controls (WT). Heat map consists of genes differentially expressed (fold change >2, adjusted *P*-value <0.01) in *Nrde2*-KO cells. Genes are divided into four clusters depending on expression changes in *Nrde2*-KO and *Mtrex*-KO samples. (*B*) Scatter plot comparing differential gene expression in *Nrde2*-KO and *Mtrex*-KO cells relative to the corresponding WT cells. Each dot represents a single gene. Genes up-regulated in 2C-like cells (Macfarlan et al. 2012) are highlighted in red. (*C*) Metaplots of normalized NRDE2, CCDC174, and EIF4A3 BCLIP-seq signal around 5′SSs of introns up-regulated (solid line) or not up-regulated (dashed line) in *Nrde2*-KO cells compared with WT. (*D*) Overlap of NRDE2 target introns (introns with a NRDE2 BCLIP-seq peak at their 5′SS) up-regulated in *Nrde2*-KO, *Ccdc174-dTAG*, or *Mtrex*-KO cells. Note that the total number of introns up-regulated might differ depending on whether protein depletion (*Ccdc174-dTAG*) or conditional gene knockout strategies were used (*Nrde2*-KO, *Mtrex*-KO). (*E*) Density plots of the distribution of NRDE2 target introns up-regulated (blue line) or not up-regulated (gray line) in *Nrde2*-KO cells based on their 5′SS, 3′SS, and branch point strength. *P*-values were calculated using the Wilcoxon rank-sum test. (*F*) Differential expression (RNA-seq) of genes containing NRDE2 target introns in *Nrde2*-KO cells (4 d of 0.1 µM 4OHT treatment) untreated (*left*) or treated (*right*) for 4 h with 100 µg/mL CHX. Genes are split into two groups: those containing only target introns not up-regulated (gray) and those containing at least one target intron up-regulated in *Nrde2*-KO cells untreated (blue) or treated with CHX (purple). *Bottom* boxplots show differential ribosome occupancy for the same genes in *Nrde2*-KO cells relative to WT. *P*-values were calculated using the Wilcoxon rank-sum test. (*G*) UCSC genome browser screenshot showing normalized BCLIP-seq, RNA-seq, and Ribo-seq tracks over *Dnmt1* gene locus. Position of the NRDE2 target intron 10 is highlighted in red.

RNA-sequencing (RNA-seq) analysis of *Nrde2*-KO cells revealed a large group of differentially expressed genes (Fig. 3A). NRDE2Δ200-expressing cells showed similar, but lower-magnitude expression changes, indicating that NRDE2 function is compromised but not abolished by the amino-terminal truncation, consistent with the viability of *Nrde2*Δ*200* cells. Most transcripts down-regulated in *Nrde2*-KO cells were also less abundant in CCDC174-depleted cells (*Ccdc174-dTAG*), whereas a large group of transcripts up-regulated in *Nrde2*-KO cells were also up-regulated upon conditional *Mtrex* knockout. These were highly enriched for genes defining a subpopulation of mESCs that resemble the gene expression state of two-cell (2C) embryos (Fig. 3B; Macfarlan et al. 2012). The expression of 2C genes correlates with the activity of MERVL, a family of retrotransposons whose expression in mESCs is regulated by facultative heterochromatin (fHC). The NRDE2 homologs in *Caenorhabditis elegans* and *Schizosaccharomyces pombe* have been linked with fHC regulation (Guang et al. 2010; Lee et al. 2013), and MERVL and its long terminal repeat (LTR) promoter, MT2_Mm, were the most up-regulated repetitive elements in both *Nrde2-*KO and *Mtrex-*KO cells (Supplemental Fig. S5A).

To test the hypothesis that NRDE2 acts with MTREX to negatively regulate MERVL expression, we created cells with an MT2_Mm-driven mNeonGreen (2C reporter) inserted in a genomic locus replacing an active MERVL (Supplemental Fig. S5B). When combined with knockouts of fHC-regulating enzymes G9A and KDM1A (Macfarlan et al. 2011, 2012), *Nrde2* knockout had an additive effect on MERVL and 2C gene up-regulation, as a consequence of the increased ratio of 2C reporter-positive cells (Supplemental Fig. S5C,D). These results indicate that NRDE2 influences MERVL expression independently of fHC formation. NRDE2 could be regulating MERVL at a post-transcriptional level, given its link to MTREX and the nuclear exosome. However, reducing the MT2_Mm reporter to shorter variants that preserve promoter activity but ultimately lead to reporter transcripts devoid of MERVL sequences did not alleviate NRDE2 dependence (Supplemental Fig. S5E,F). Therefore, the observed up-regulation of MERVL and 2C genes upon *Nrde2* or *Mtrex* knockout likely represents a pleiotropic effect caused by altered RNA metabolism, rather than an escape from the specific action of NRDE2 and MTREX on these genes. This conclusion is further supported by the negligible effects of the NRDE2-D174R mutation on gene expression, including 2C genes (Fig. 3A; Supplemental Fig. S5G).

### NRDE2 and CCDC174 regulate weak 5′ splice sites

Given NRDE2 and its interactor CCDC174 bind to 5′SSs, we hypothesized that pre-mRNA splicing could be defective in mutant cell lines, manifest as globally increased intronic signals in the RNA-seq data. To exclude transcription activation effects, we analyzed down-regulated transcripts only (log_2_ fold change <0). In both *Nrde2-*KO and *Ccdc174-dTAG* cells, we found hundreds of misspliced up-regulated introns that were generally enriched for NRDE2 and CCDC174 binding at their 5′SSs (Fig. 3C; Supplemental Fig. S6A). However, the up-regulated introns constituted only a small fraction of all the introns defined as NRDE2 and CCDC174 target introns (5.8% and 2%, respectively), for which the peak-calling analysis of the BCLIP-seq data identified a NRDE2 or CCDC174 peak at their 5′SS. The majority of the target introns with NRDE2- or CCDC174-bound 5′SSs remained unaffected. Misspliced target introns largely overlapped in the *Nrde2-*KO and *Ccdc174-dTAG* data sets, but they were distinct from target introns up-regulated in *Mtrex-*KO cells (Fig. 3D; Supplemental Fig. S6B). Hence, ablation of NRDE2 or CCDC174 can have a direct effect on the splicing of a subset of introns they associate with, which is not observed for MTREX. Therefore, NRDE2 and CCDC174 likely function independently of MTREX in pre-mRNA splicing. We note that the number of NRDE2-bound up-regulated introns was much smaller in *Nrde2*Δ*200* compared with *Nrde2*-KO cells, suggesting the amino-terminally truncated NRDE2 functions as a hypomorph (Supplemental Fig. S6C).

Our observation that splicing of only a minor fraction of NRDE2 and CCDC174-bound introns was affected in *Nrde2-*KO cells could be due in part to the limited sensitivity of RNA-seq for detecting unstable misspliced transcripts. These could be rapidly degraded cotranscriptionally and/or by translation-dependent RNA surveillance in the cytoplasm (Gordon et al. 2021). Whereas testing the former possibility is not straight-forward, we tested the latter by blocking translation-dependent RNA surveillance by cycloheximide (CHX) treatment. This led to an increase in misspliced introns (12% of NRDE2-bound and 17% of CCDC174-bound) that overlapped between different conditions (Supplemental Fig. S6C). The majority of introns remained unchanged even after CHX treatment, prompting us to investigate the selective sensitivity of target introns to NRDE2 or CCDC174 depletion. Analysis of intron features revealed that NRDE2-bound introns up-regulated in *Nrde2-*KO cells possess weaker 5′SSs, while their 3′SSs and branch points are comparable with nonaffected introns (Fig. 3E). The difference in 5′SS strength was more pronounced in the group of introns up-regulated in CHX-treated *Nrde2*-KO cells. The same was observed for *Ccdc174*-bound introns (Supplemental Fig. S6D,E).

The aberrant splicing of introns in *Nrde2-*KO and *Ccdc174-dTAG* cells resulted in an overall decrease of the respective mRNA signal (Fig. 3F; Supplemental Fig. S6F). However, the impact on mRNA levels was generally small, possibly due to the stability of the aberrantly spliced transcripts. To assess the effect on translation, we performed ribosome profiling (Ribo-seq) in wild-type and *Nrde2-*KO cells. Compared with RNA-seq, a larger Ribo-seq signal reduction was observed for messages whose splicing was affected by *Nrde2* knockout (Fig. 3F). For example, intron 10 of the *Dnmt1* pre-mRNA has a suboptimal 5′SS that is bound by NRDE2 and CCDC174. Intron 10 levels were up-regulated in *Nrde2-*KO and CCDC174-depleted cells without a substantial effect on mRNA levels, whereas ribosome occupancy was markedly reduced (Fig. 3G; Supplemental Fig. S6G). Thus, overall, NRDE2 and CCDC174 are required for efficient splicing of a largely overlapping set of target introns with suboptimal 5′SS sequences.

### NRDE2 is necessary for correct splice site choice

**FIGURE 4.**
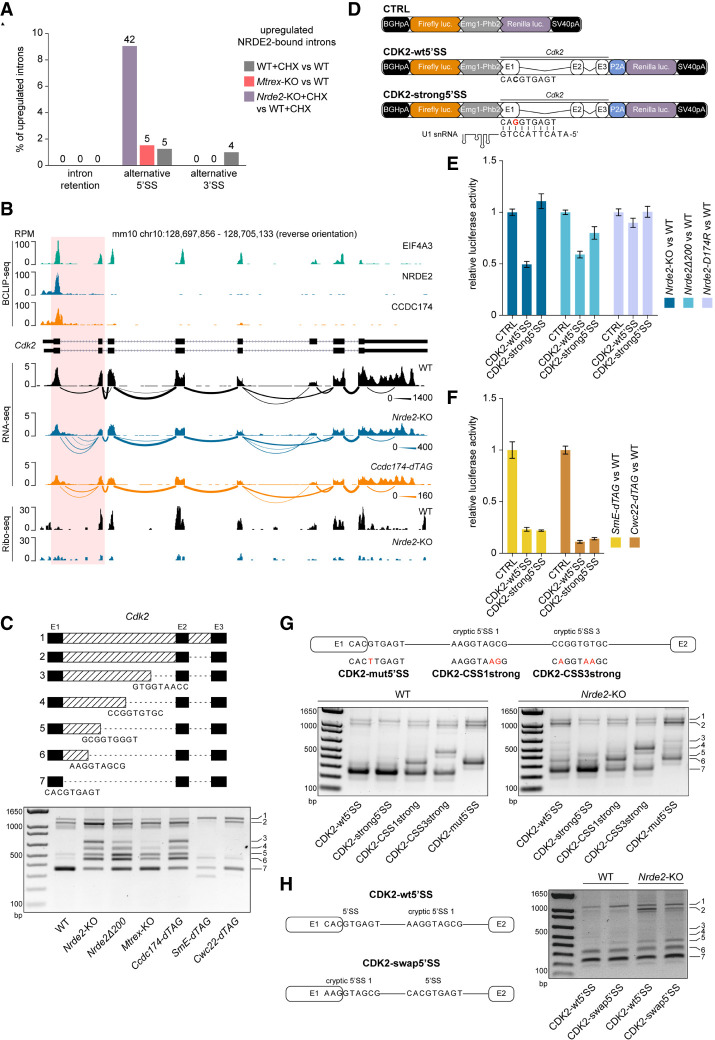
NRDE2 regulates 5′SS selection and splicing of its target gene Cdk2. (*A*) rMATS analysis (Shen et al. 2014) of alternative splicing events within NRDE2 target introns up-regulated (inclusion level difference >0.1) in *Mtrex*-KO cells and in CHX-treated WT and *Nrde2*-KO cells relative to their corresponding WT and untreated controls (only introns with more than 30 reads across all samples were included in the analysis). (*B*) UCSC genome browser screenshot showing normalized BCLIP-seq, RNA-seq, and Ribo-seq tracks over *Cdk2* gene locus combined with a Sashimi plot of *Cdk2* splicing patterns detected in the RNA-seq data. The thickness of the splice junction connecting lines reflects the number of corresponding spliced RNA-seq reads with a scale provided at the *right*. NRDE2 and CCDC174 target intron 1 is highlighted in red. (*C*) RT-PCR analysis of *Cdk2* intron 1 splicing with primers binding in exons 1 and 3. The additional bands in *Nrde2*-KO sample were sequenced and the identified cryptic 5′SSs are depicted in the scheme at the *top*. (*D*) Schematic overview of the CDK2 dual-luciferase reporters. The point mutation introduced in *Cdk2* intron 1 5′SS to improve U1 snRNA complementarity in the CDK2-strong5′SS reporter is depicted in red. (*E*,*F*) Dual-luciferase assay of the transiently transfected CDK2 reporters. Plotted is the average value of normalized Renilla luciferase activity from three independent experiments each performed in triplicate transfections. Error bars represent standard deviation. (*G*) RT-PCR analysis of CDK2 reporter splicing in transiently transfected WT and *Nrde2*-KO cells with primers binding in *Cdk2* exon1 and in the P2A peptide region. The additional mutations introduced in the CDK2-wt5′SS reporter are depicted in red. The band numbering at the *right* corresponds to the scheme in *C*. (*H*) Same as in *G*, comparison of splicing in CDK2-wt5′SS reporter and CDK2-swap5′SS with the annotated 5′SS and the first cryptic 5′SS swapped, as depicted in the scheme.

Defective splicing can lead to intron retention, splicing from a cryptic 5′SS, or splicing to a cryptic 3′SS. Analysis of novel splicing events in up-regulated NRDE2-bound introns revealed frequent cryptic 5′SS usage upon *Nrde2* knockout (Fig. 4A). Likewise, CCDC174-bound up-regulated introns showed higher incidence of cryptic 5′SS usage upon CCDC174 depletion (Supplemental Fig. S7A). The first intron of the essential cell cycle regulator gene *Cdk2*, which contains a weak 5′SS strongly bound by NRDE2 and CCDC174, provides a representative example of such aberrant splice site choice. Examination of our RNA-seq data uncovered a shift in *Cdk2* intron 1 splicing from the annotated 5′SS to several cryptic 5′SSs in cells lacking NRDE2 or CCDC174 (Fig. 4B). Splicing from the cryptic 5′SSs generated nonfunctional transcripts because of an in-frame stop codon immediately downstream from the first annotated 5′SS, resulting in a substantial reduction in Ribo-seq signal (Fig. 4B). Usage of multiple alternative cryptic 5′SSs was confirmed by RT-PCR with primers binding to *Cdk2* exons 1 and 3 (Fig. 4C). We observed a similar but weaker aberrant splicing pattern in NRDE2Δ200-expressing cells. Usage of a small number of cryptic 5′SSs was also detected in *Mtrex-*KO cells, likely due to compromised NRDE2 stability. In contrast, depletion of the general splicing factors SmE (a component of the snRNP-stabilizing Sm ring complex) or CWC22 resulted in minimal cryptic 5′SS usage. Notably, all the alternative 5′SSs in the *Cdk2* intron 1 that are used in the absence of NRDE2 or CCDC174 consist of suboptimal sequences. Thus, NRDE2 and CCDC174 appear to be dispensable for general splicing, but are required for appropriate splice site choice.

To determine whether NRDE2 splice site choice is modulated by 5′SS strength and to validate *Cdk2* intron 1 as a NRDE2 target, we fused *Cdk2* exons 1–3 with the *Renilla* luciferase gene in a dual-luciferase reporter plasmid (CDK2-wt5′SS). We also generated a CDK2 reporter construct in which the sequence of the first 5′SS was optimized by a single point mutation (CDK2-strong5′SS), improving U1 snRNA complementarity (Fig. 4D). Compared with wild-type cells, the activity of CDK2-wt5′SS was twofold lower when expressed in *Nrde2-*KO cells, whereas CDK2-strong5′SS was insensitive to NRDE2 deficiency (Fig. 4E). Thus, 5′SS strength is a predictive feature of NRDE2-dependent CDK2 pre-mRNA splicing.

CDK2-wt5′SS activity was insensitive to the NRDE2-D174R mutation, but was affected by the amino-terminal truncation (NRDE2Δ200) (Fig. 4E), further supporting the idea that NRDE2 functions independently of MTREX in pre-mRNA splicing, and that the amino terminus of NRDE2 contributes to its splicing-related activity. In comparison with *Nrde2-*KO cells, the reduction of CDK2 reporter activity was more substantial in cells lacking SmE or CWC22, regardless of 5′SS strength (Fig. 4F), indicating that NRDE2 is not an obligate splicing factor, but is necessary for the efficient use of weak 5′SSs. To support this conclusion, we generated additional reporter constructs containing NRDE2-bound sequences of the DNA damage response regulator *Tti1* gene, where NRDE2 and CCDC174 bind the 5′SS of intron 5 (Supplemental Fig. S7B,C). A 2-nt substitution that strengthened the weak 5′SS of intron 5 abolished the NRDE2-dependency of *Tti1* splicing (Supplemental Fig. S7D–F), consistent with the CDK2 reporters.

The first *Cdk2* intron harbors multiple cryptic 5′SSs, which allowed us to dissect the 5′SS choice hierarchy. We introduced several mutations into the CDK2-wt5′SS luciferase reporter: mutations that improve the strength of either the first or third cryptic 5′SS, or a mutation that inactivates the annotated 5′SS (Fig. 4G). As predicted by our model, the lack of NRDE2 resulted in the usage of four additional cryptic 5′SSs located downstream from the annotated 5′SS. The shift in splice site choice upon *Nrde2* knockout was diminished if the annotated 5′SS was mutated to a strong 5′SS. Increasing the strength of the first or third cryptic 5′SS resulted in their usage in wild-type cells, along with the annotated 5′SS. This balance shifted toward the strengthened cryptic 5′SSs in *Nrde2-*KO cells, indicating that NRDE2 enhances splicing from the most upstream weak 5′SS, even when followed by a stronger downstream 5′SS. Notably, inactivation of the annotated 5′SS redirected NRDE2 regulation to the neighboring downstream cryptic 5′SS, which was the exclusive 5′SS used in wild-type cells. In the absence of NRDE2, the remaining cryptic sites were chosen again (Fig. 4G). Furthermore, swapping the annotated 5′SS and the first cryptic 5′SS in the CDK2 luciferase reporter did not affect the splicing pattern either in wild-type or in *Nrde2*-KO cells (Fig. 4H). Thus, NRDE2 promotes splicing from the most upstream of a series of 5′SSs that we have tested. Before generalizing this conclusion, additional NRDE2-responsive pre-mRNAs should be investigated.

### CCDC174 function depends on NRDE2

**FIGURE 5.**
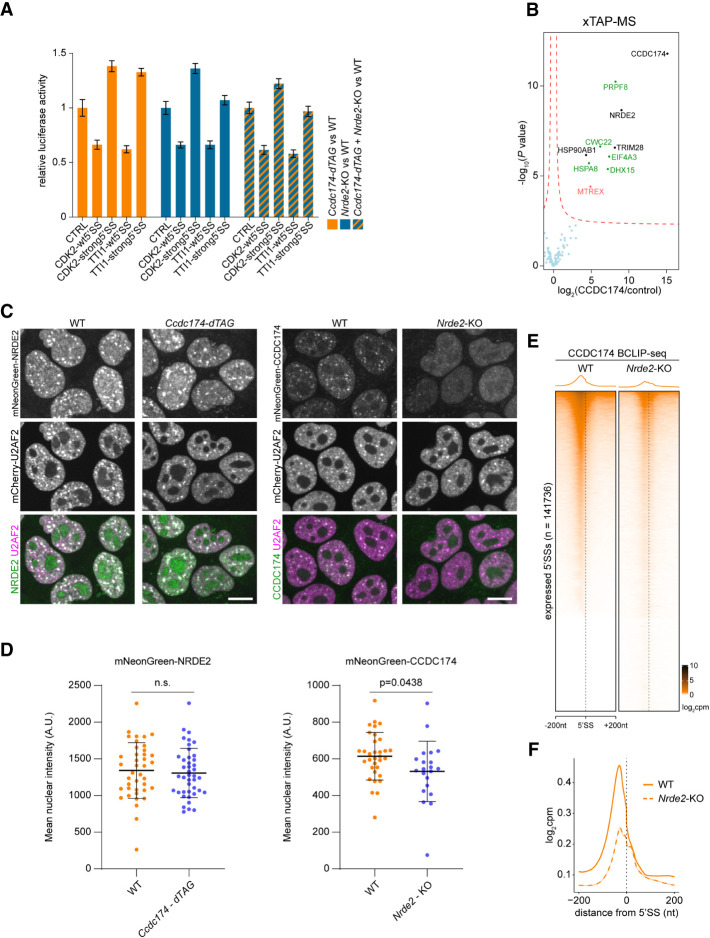
NRDE2 is required for the proper recruitment of CCDC174 to 5′SSs. (*A*) Dual-luciferase assay of the transiently transfected CDK2 and TTI1 reporters in cells allowing simultaneous depletion of NRDE2 and CCDC174. Plotted is the average value of normalized Renilla luciferase activity from three independent experiments each performed in triplicate transfections. Error bars represent standard deviation. (*B*) Formaldehyde cross-linking TAP-MS analysis of 3A-CCDC174 compared with untagged control and performed in three independent replicates for each sample. Same labeling scheme as in Figure 1, A and B, applies. (*C*) Live-cell spinning-disk microscopy of endogenous mNeonGreen-tagged NRDE2 and CCDC174 with endogenous mCherry-tagged U2AF2 in cells allowing depletion of CCDC174 and NRDE2, respectively. Scale bar 10 µm. (*D*) Quantification of live-cell spinning-disk microscopy fluorescence signal intensity for endogenously tagged mNeonGreen-NRDE2 in WT and CCDC174-depleted cells, and mNeonGreen-CCDC174 in WT and *Nrde2*-KO cells. Each dot represents a single nucleus. Statistical significance was assessed by unpaired two-tailed *t*-test. (*E*) Heat maps of the normalized CCDC174 BCLIP-seq signal intensity in WT and *Nrde2*-KO cells centered at annotated 5′SSs of all transcripts expressed in mESCs. Splice sites are ordered by decreasing CCDC174 signal in the WT sample. (*F*) Metaplots of normalized CCDC174 BCLIP-seq signal around all annotated expressed 5′SSs in WT (solid line; same data as in Fig. 2D) and *Nrde2*-KO (dashed line).

NRDE2 and CCDC174 promote splicing at many of the same weak 5′SSs, with, for example, the activity of the CDK2 and TTI1 luciferase reporters being the same in *Nrde2-*KO and *Ccdc174-dTAG* cells (Fig. 5A). Simultaneous depletion of NRDE2 and CCDC174 had no additive effect on reporter activity, suggesting that the two proteins act together. This was further supported by xTAP-MS of CCDC174, which identified a set of splicing factors similar to those present in the NRDE2 interactome (Figs. 1C, 5B). CCDC174 also accumulated in NSs, and its accumulation was reduced by *Nrde2* knockout, while the overall level was only minimally decreased (Fig. 5C,D). NRDE2 level and NS localization remained unchanged in CCDC174-depleted cells (Fig. 5C,D). Hence, NRDE2 recruits CCDC174 to NSs to exert its splicing-related function.

To analyze the relationship between NRDE2 and CCDC174, we performed CCDC174 BCLIP-seq in *Nrde2-*KO cells. Overall binding of CCDC174 at 5′SSs was substantially reduced in the absence of NRDE2 (Fig. 5E). Interestingly, the lack of NRDE2 mainly affected CCDC174 binding in the exonic region upstream of 5′SSs, whereas the average low-level intronic CCDC174 BCLIP-seq signal remained comparable between wild-type and *Nrde2*-KO cells (Fig. 5F). Residual CCDC174 binding to 5′SSs could therefore persist in the absence of NRDE2, yet at a different preferred position. Taken together, these results demonstrate that NRDE2 is required for efficient recruitment of CCDC174 to NSs and association with 5′SSs, where the two proteins cooperate to sustain weak 5′SS selection and pre-mRNA splicing.

### NRDE2 and CCDC174 bind to U1 snRNA

**FIGURE 6.**
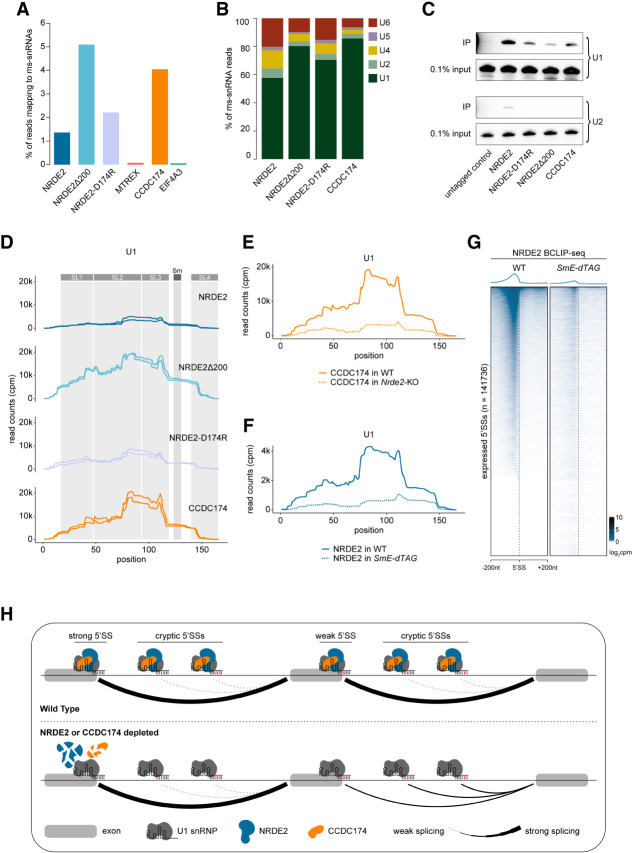
NRDE2 and CCDC174 bind to U1 snRNA. (*A*) Proportion of BCLIP-seq reads mapping to ms-snRNAs. (*B*) Relative distribution of ms-snRNA-mapping BCLIP-seq reads among individual snRNAs. (*C*) Northern blotting analysis of U1 and U2 snRNAs in immunoprecipitates of tandem affinity purified control untagged cells and indicated endogenously 3A-tagged cells. (*D*) BCLIP-seq signal coverage over U1 snRNA sequence (including reads mapping to U1a1, U1b1, and U1b6 isoforms) showing the two BCLIP-seq replicates of each sample separately. Gray columns indicate the positions of the Sm ring binding site and the four stem–loops within the U1 snRNA secondary structure. (*E*) CCDC174 BCLIP-seq signal coverage over U1 snRNA sequence (including reads mapping to U1a1, U1b1, and U1b6 isoforms) in WT and *Nrde2*-KO cells. (*F*) NRDE2 BCLIP-seq signal coverage over U1 snRNA sequence (including reads mapping to U1a1, U1b1, and U1b6 isoforms) in WT and *SmE-dTAG* cells. (*G*) Heat maps of the normalized NRDE2 BCLIP-seq signal intensity in WT and *SmE-dTAG* cells centered at annotated 5′SSs of all transcripts expressed in mESCs. Splice sites are ordered by decreasing NRDE2 signal in the WT sample, which is the same as in Figure 2C. (*H*) Schematic summary of the presented data showing that NRDE2 and CCDC174 associate with U1 snRNA to indiscriminately bind both strong and weak 5′SSs. Whereas depletion of either NRDE2 or CCDC174 has no effect on splicing strong 5′SSs, it leads to reduced splicing of weak 5′SSs with concomitant increase in intronic cryptic 5′SS usage.

Participation of NRDE2 in 5′SS choice raises the question how 5′SSs are recognized by NRDE2. Furthermore, how does NRDE2 recruit CCDC174, given that they interact weakly in an RNA-dependent manner? Considering NRDE2Δ200 localized to NSs and was partially functional, despite losing interactions with splicing factors and no longer cross-linking to pre-mRNAs, it seemed unlikely that NRDE2 engages with the spliceosome via interaction with one of its proteinaceous components or by binding to a specific sequence motif in pre-mRNA. Instead, the NRDE2 BCLIP-seq data sets were highly enriched for reads mapping to major spliceosome snRNAs (ms-snRNAs), an association that is highly specific, given we did not observe this for MTREX or EIF4A3 (Fig. 6A). The portion of ms-snRNA-mapping reads further increased in NRDE2-D174R libraries and reached maximal levels in NRDE2Δ200 samples (5% of total reads). CCDC174 was equally strongly associated with ms-snRNAs (4% of total reads). No appreciable enrichment in signal was observed for minor spliceosome snRNAs. The majority of ms-snRNA reads in all NRDE2 and CCDC174 samples mapped to U1 snRNA, from nearly 60% for NRDE2 to more than 80% for NRDE2Δ200 and CCDC174 (Fig. 6B). The association of NRDE2 and CCDC174 with U1 snRNA was confirmed under native conditions by immunoprecipitation followed by northern blotting (Fig. 6C). In addition, both NRDE2 and CCDC174 coimmunoprecipitated with an anti-m3G antibody, which recognizes the trimethylated guanosine cap of snRNAs, in an RNase-sensitive manner (Supplemental Fig. S8A). The BCLIP-seq signal covered most of the highly structured U1 snRNA sequence, with the highest enrichment over the stem–loops II and III (SL2 and SL3) for both NRDE2 and CCDC174 (Fig. 6D), indicating that NRDE2 and CCDC174 contact U1 snRNA at multiple positions. In comparison, the two other most represented ms-snRNAs, U4 and U6, appeared to cross-link at distinct locations overlapping the U4/U6 stem III region (Supplemental Fig. S8B). U1 SL2 and SL3 and U4/U6 stem III are in close proximity in the precatalytic spliceosomal pre-B complex (Supplemental Fig. S8C; Charenton et al. 2019), suggesting how U1 snRNA-bound NRDE2 and CCDC174 might contact U4 and U6 snRNAs. Together, these results reveal that U1 snRNA is the primary RNA bound by NRDE2. Given CCDC174 association with U1 snRNA was reduced in the absence of NRDE2 (Fig. 6E), we conclude that CCDC174 preferably binds NRDE2-bound U1 snRNAs.

The indiscriminate association of NRDE2 and CCDC174 with 5′SSs is explained by U1 snRNA-mediated targeting. To further test this model, we decided to perturb the structure of the Sm ring complex, which is necessary for U1 snRNA maturation and trafficking (Matera and Wang 2014). Depletion of the Sm ring component SmE led to a significant reduction of NRDE2 BCLIP-seq signal on U1 snRNA (Fig. 6F), demonstrating that NRDE2 association with U1 snRNA depends on a functional U1 snRNP assembly pathway. Consistent with U1 snRNA-mediated targeting of NRDE2 to pre-mRNAs, NRDE2 association with 5′SSs was substantially reduced upon SmE depletion (Fig. 6G). Therefore, we propose a model in which NRDE2 and CCDC174 are directly recruited by U1 snRNA to sustain the splicing of weak 5′SSs.

## DISCUSSION

Mammalian cells must cope with the challenge of selecting and splicing 5′SSs with suboptimal U1 snRNA complementarity. Mechanistic studies on weak 5′SS splicing have thus far focused on auxiliary splicing factors, mainly from the SR-protein family, that bind exonic splicing enhancer (ESE) motifs in the vicinity of 5′SSs to support the binding of canonical U1 snRNPs (Wu and Maniatis 1993; Kohtz et al. 1994; Long and Caceres 2008). Our study uncovers an alternative strategy that involves the conserved proteins NRDE2 and CCDC174 interacting directly with U1 snRNA to promote the selection and splicing of weak 5′SSs (Fig. 6H).

### NRDE2 and CCDC174 bind RNA

The composition of the NRDE2 and CCDC174 PPI networks links these two proteins to RNA metabolism, raising the question whether they have intrinsic RNA-binding activities. RNA binding is commonly probed using CLIP, which enriches for RNAs in direct contact with the protein of interest due to zero-distance UV cross-linking (Lee and Ule 2018). When using existing CLIP protocols for NRDE2 and unrelated low-abundant proteins, we suffered from undesirable sample loss. Speculating that we might lose our samples because of the low UV cross-linking efficiency (Hafner et al. 2021) and during the many steps required to purify the RNA–protein complexes, we designed a simplified BCLIP-seq protocol that uses high-affinity 3A tag components. This substantially reduced length and complexity of the cross-linked protein–RNA purification. The protocol is further streamlined by a ligation-free sequencing library construction using the template-switching activity of SuperScript II reverse transcriptase. Because this relies on the presence of Mn^2+^ ions, the resulting library might lack the single-nucleotide resolution of the UV cross-linked nucleotide as suggested previously (Nostrand et al. 2017). Although neither NRDE2 nor CCDC174 contains predicted RNA-binding domains, our BCLIP-seq results revealed that both of them bind to RNA. Thus, NRDE2 and CCDC174 belong to a group of proteins whose amino acid sequences are not predictive of RNA-binding activity (Albihlal and Gerber 2018).

### NRDE2 and CCDC174 modulate weak 5′SSs

Our results revealed that the amino terminus of NRDE2 is dispensable for U1 snRNA binding, but is necessary for associating with pre-mRNAs at 5′SSs. This highlights U1 snRNA as the primary RNA bound by NRDE2 and offers insights into the potential mechanisms that stabilize the association of the U1 snRNP with weak 5′SSs. It is possible that the amino terminus of NRDE2 mediates stabilizing interactions within the spliceosome once bound to the 5′SS. The amino terminus could also interact with the pre-mRNA, further stabilizing U1 snRNA association with weak 5′SS. Additional work will be required to fully elucidate these mechanisms, but such model may help explain the hypomorphic phenotype of the NRDE2Δ200 truncation.

Our results are also relevant for understanding splice site choice. The luciferase reporter experiments revealed that NRDE2 stimulates splicing from the most upstream 5′SS of an intron, reminiscent of a previously described function of hnRNPA1 in in vitro splicing reactions (Mayeda and Krainer 1992). In a cotranscriptional splicing model, this could simply be explained by the fact that the most upstream 5′SS emerges from the RNA polymerase first. The results we have obtained with the CDK2 reporter constructs support this hypothesis (Fig. 4G,H). Alternatively, it could be mediated by proteins binding to upstream ESEs. Indeed, we found several ESE-binding SR proteins interacting with NRDE2, but not with NRDE2Δ200 (Fig. 1A,B) for which the most upstream 5′SS preference is partially lost. The luciferase reporter assays also revealed that NRDE2 participates in the splicing of target introns, rather than recruiting MTREX to degrade unspliced or incorrectly spliced transcripts, as suggested for the NRDE2 homolog in *S. pombe* (Zhou et al. 2015). Therefore, the function of the NRDE2-MTREX interaction in mammalian cells remains elusive. MTREX is required for NRDE2 stability, and based on our xTAP-MS results, it retains contact with NRDE2-D174R and NRDE2Δ200. Given its RNA helicase activity, a possible MTREX function could be unwinding of the NRDE2-bound, highly structured U1 snRNA. In this model, the arch interacting motif within the NRDE2 amino terminus would stabilize MTREX association to allow complete RNA unwinding.

### NRDE2 and CCDC174 are part of the major spliceosome

Our proteomic data and the BCLIP-seq signals at unspliced 5′SSs indicate that NRDE2 and CCDC174 associate with spliceosome components during splicing. However, they have not been identified in spliceosome composition studies (Rappsilber et al. 2002; Zhou et al. 2002; Wahl et al. 2009). Such analyses, both at the proteomic and structural level, have relied on spliceosomes preassembled on model pre-mRNA substrates with optimal splicing features, suggesting that they may not reflect the full scope of in vivo spliceosome diversity. In structural studies, the spliceosome behaves as a dynamic assembly progressing through distinct precatalytic, catalytic, postcatalytic, and disassembly steps accompanied by considerable rearrangements, including U1 snRNP dissociation before the catalytic step (Wilkinson et al. 2019). Our data demonstrate simultaneous NRDE2 and CCDC174 association with the precatalytic spliceosome, represented by binding to U1 snRNA and unspliced 5′SSs, and with the late postcatalytic spliceosome via interactions with disassembly factors DHX15 and TFIP11. Hence, we speculate that NRDE2 and CCDC174 may be involved in an alternative spliceosome assembly pathway.

Notably, NRDE2 interactions with splicing factors were revealed by nTAP-MS only after digesting the samples with Benzonase nuclease, which loosens large RNP assemblies. Moreover, core spliceosome components and disassembly factors, as well as CCDC174, only appeared in xTAP-MS under harsh denaturing conditions, and freeze-thawing the purified xTAP-MS samples on magnetic beads caused a reduction in detected splicing factors, whereas the recovery of MTREX was not affected. These observations all hint at the physical properties of NRDE2-associated spliceosomes that make detection difficult in ordinary workflows, such that they have remained unnoticed.

In conclusion, we present evidence for a splicing mechanism that has evolved to effectively select and splice weak 5′SSs in hundreds of genes in mESCs. Investigations of its structural and functional properties, cell type specificity, and evolutionary conservation will further our understanding of pre-mRNA splicing regulation, both in healthy and diseased states.

## MATERIALS AND METHODS

For reagents and resources, see Table 1.

**TABLE 1. RNA079465FLETB1:**
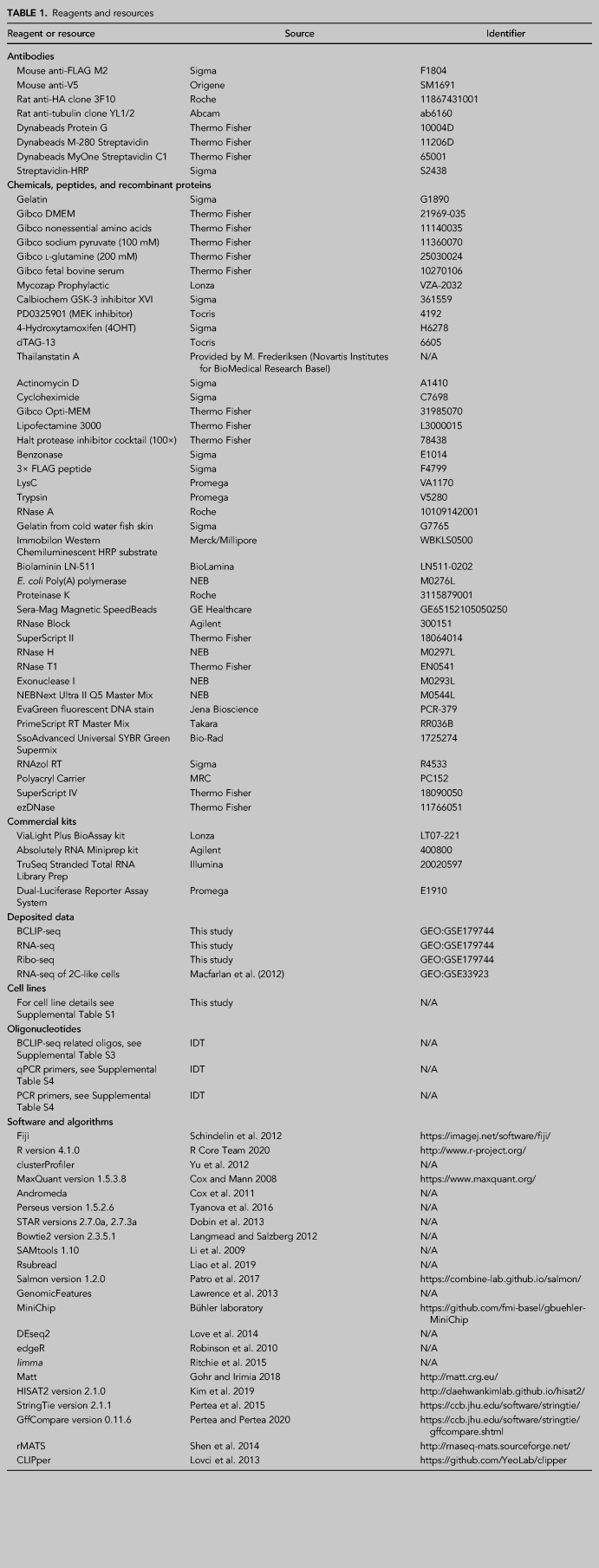
Reagents and resources

### Experimental model and subject details

Male mESCs of a mixed 129xC57BL/6 background with a heterozygous integration of only Cre-ERT2 (cMB052) or both Cre-ERT2 and V5-tagged BirA (cMB063) in the *Rosa26* locus (Flemr and Bühler 2015; Ostapcuk et al. 2018) were used as parental lines to generate all the genome-edited cell lines used in this study.

### Cell culture and treatment

Unless stated otherwise, cells were grown on gelatin-coated dishes in S-L-2i medium (DMEM [Gibco] supplemented with 15% fetal bovine serum [Gibco], 1× nonessential amino acids [Gibco], 1 mM sodium pyruvate [Gibco], 2 mM l-glutamine [Gibco], 0.1 mM 2-mercaptoethanol [Sigma], 50 µg/mL penicillin, 80 µg/mL streptomycin, 1:500 MycoZap Prophylactic [Lonza], homemade LIF conditioned medium, and 2i inhibitors: 3 µM GSK-3 inhibitor XVI [Sigma] and 10 µM MEK inhibitor PD0325901 [Tocris]). To induce conditional knockout of *Nrde2* and *Mtrex*, 4OHT (Sigma; 1:5000 dilution of 0.5 mM stock dissolved in ethanol) was added to the medium and replaced daily for a total of 4 and 3 d, respectively. Depletion of endogenous proteins fused to the 2xHA-FKBP12^F36V^ domain (dTAG) was achieved by treating the cells with 0.5 µM dTAG-13 compound (Tocris; 1:1000 dilution of 0.5 mM stock solution in DMSO) for 24 h. For chemical inhibition of splicing, cells were treated with 1 µM Thailanstatin A (provided by P. Krastel and M. Frederiksen from Novartis Institutes for BioMedical Research; 1:1000 dilution of 1 mM stock solution in DMSO) for 6 h. To block transcription, cells were treated with 5 µg/mL Actinomycin D (Sigma; 1:1000 dilution of 5 mg/mL stock solution in DMSO) for 2 h. For translation inhibition, 100 µg/mL Cycloheximide (Sigma; 1:1000 dilution of 100 mg/mL stock solution in DMSO) was added for 4 h.

### Genome editing

We used both TALEN and Cas9 nucleases to generate cell lines with endogenous gene modifications. Endogenous *Nrde2* truncation and point mutation, tagging with 3A tag and loxP site insertions were achieved using single-stranded oligodeoxynucleotides with short homology arms synthesized by Integrated DNA Technologies. Longer inserts flanked by longer homology arms were cloned into pBluescript II KS-plasmid (Agilent Technologies). All TALEN and Cas9 target sequences and donor sequences for homologous recombination are listed in Supplemental Table S1. Cells grown in medium without 2i inhibitors were seeded 5 × 10^5^ per well of a six-well plate and transfected with a mixture of 500 ng of donor DNA and 500 ng of Cas9-2A-Puro/TALEN mix with pRR-Puro reporter (Flemr and Bühler 2015) in Opti-MEM medium (Gibco) using Lipofectamine 3000 (Thermo Fisher Scientific), according to the manufacturer's instructions. The transfected cells were selected by a 24-h treatment with 2 µg/mL puromycin (Sigma) and expanded at a clonal density, and the clones were picked and genotyped by PCR. Correct homozygous insertions were verified by Sanger sequencing.

### Growth curve

Cell growth was monitored using the ViaLight Plus Cell Proliferation and Cytotoxicity BioAssay kit (Lonza). Triplicates of 500 cells in 0.5 mL of medium were seeded on a 24-well plate. After 24 h, the medium was removed from the wells of day 0 time point and replaced with 50 µL of PBS. Next, 10 µL of ViaLight cell lysis reagent was added and mixed by pipetting, and the plate was incubated on a rocking platform for 10 min at room temperature. An amount of 10 µL of the lysate was mixed with 10 µL of AMR reagent in a 96-well solid-bottom white assay microplate (Corning) and, after 2-min incubation at room temperature, the luminescence was measured in Mithras LB 940 microplate reader (Berthold Technologies). In the wells of later time points, the medium was replaced 24 h after seeding and every following 24 h with fresh medium with or without 0.1 µM 4OHT. All the later time points were processed for luminescence measurement as described above.

### nTAP-MS of 3A-tagged proteins

Each nTAP-MS was performed in three independent replicates. Three near-confluent 10-cm dishes (∼5 × 10^7^ cells) were used per each replicate sample. The dishes were placed on ice, and cells were rinsed with cold PBS and collected by scraping and spinning at 3000*g* for 2 min at 4°C. The pellets were washed with 0.5% BSA in PBS and then resuspended in 0.5 mL of ice-cold B100 buffer (10 mM Tris-HCl at pH 7.5, 2 mM MgCl_2_, 100 mM NaCl, 0.5% Triton X-100) supplemented with 1× Halt Protease Inhibitor Cocktail (HALT) (Thermo Fisher Scientific) and 100 U of Benzonase (Sigma). After 30-min shaking (500 RPM) at 12°C, lysates were diluted with 450 µL of B100 + HALT and centrifuged at 16,000*g* for 5 min at 4°C.

For each sample, Protein G Dynabeads (Thermo Fisher Scientific) equivalent to 20 µL of the original slurry were resuspended in PBS with 0.02% Tween-20 and coupled with 2 µL of anti-FLAG M2 antibody (Sigma), rotating for 30 min at room temperature. Coupled beads were washed once with PBS + 0.02% Tween-20 and twice with B100. The beads were then resuspended in 50 µL of B100 + HALT, mixed with the cleared lysates, and rotated for 2 h at 4°C. Following three washes with 1 mL of ice-cold B100, FLAG-tagged proteins were eluted by 15-min shaking (500 RPM) in 25 µL of B100 + HALT containing 1 mg/mL 3xFLAG peptide (Sigma) at room temperature. The beads were further rinsed with 425 µL of B100 + HALT, and the supernatant was combined with the 25 µL of eluate from the previous step.

M-280 Streptavidin Dynabeads (Thermo Fisher Scientific) equivalent to 20 µL of the original slurry were prewashed twice with B100, resuspended in 50 µL of B100 + HALT, mixed with the FLAG beads eluates, and rotated for 30 min at room temperature. The beads were then washed three times with 1 mL of B100, resuspended in 0.2 mL of TN buffer (10 mM Tris-HCl at pH 7.5, 100 mM NaCl), and transferred to a new tube. While remaining immobilized on the magnetic stand, the beads were rinsed with an additional 1 mL of TN buffer.

On-bead digestion for mass spectrometry was performed as follows. The washed detergent-free beads were resuspended by vortexing in 5 µL of digestion buffer (8 M urea freshly dissolved in 20 mM HEPES at pH 8.5, 5 mM TCEP, and 10 mM chloroacetamide) and sonicated in Bioruptor Plus (Diagenode) at high energy in 30 cycles, 10 sec ON/10 sec OFF. Next, 1 µL of 0.2 mg/mL LysC protease (Promega) in 50 mM HEPES (pH 8.5) was added, and proteins were predigested for 2 h rotating at room temperature. The urea concentration was then diluted with 17 µL of 50 mM HEPES (pH 8.5) and, after adding 1 µL of 0.2 mg/mL trypsin (Promega) in 0.2 mM HCl, the digestion continued overnight at 37°C with interval mixing at 2000 RPM for 30 sec every 15 min. Digested proteins were then subjected to mass spectrometry as described below in “Mass spectrometry analysis.”

### xTAP-MS of 3A-tagged proteins

Each xTAP-MS was performed in three independent replicates. For each replicate sample, cells were harvested by trypsinization, placed on ice, and counted on a Countess II Automated Cell Counter (Thermo Fisher Scientific), and 4.5 × 10^7^ cells were collected by centrifugation at 300*g* for 2 min and 4°C. The pellets were resuspended in 1.5 mL of room temperature PBS and cross-linking started by the addition of 1.5 mL of 0.2% formaldehyde in PBS. After 10 min rotating at room temperature, 150 µL of 2.5 M glycine was added and samples were placed for 2 min on ice to quench the excess formaldehyde. Cells were pelleted, rinsed with 0.2% BSA in cold PBS, and finally resuspended by vortexing in 180 µL of TMS buffer (10 mM Tris-HCl at pH 8.0, 1 mM MgCl_2_, 1% SDS, 1× HALT) prechilled at 12°C and supplemented with 100 U of Benzonase. Following 30-min incubation at 12°C, the lysates were diluted with 1.57 mL of DIL. mix containing a 4:5 (v/v) ratio of H_2_O and dilution buffer (20 mM Tris-HCl at pH 8.0, 1 M NaCl, 2% Triton X-100, 20 mM EDTA), supplemented with 1× HALT and incubated for 5 min on ice. Any remaining insoluble material was removed by spinning at 16,000*g* for 5 min at 4°C.

The cleared lysates were mixed with anti-FLAG antibody-coupled Protein G Dynabeads, which were prepared essentially as described above in the nTAP-MS section, but prewashed with 2 × 1 mL of WASH buffer (10 mM Tris-HCl at pH 8.0, 0.5 M NaCl, 1% Triton X-100, 0.1% SDS, 1 mM EDTA) and resuspended in 50 µL of DIL. mix. After 2 h rotating at 4°C, the beads were washed three times with 1 mL of cold WASH buffer and FLAG-tagged proteins were eluted by shaking at 500 RPM for 15 min in 25 µL of TEDS buffer (10 mM Tris-HCl at pH 8.0, 1 mM EDTA, 10 mM DTT, 1% SDS, 1× HALT) containing 1 mg/mL 3xFLAG peptide at room temperature. The beads were rinsed with another 25 µL of TEDS, and the supernatant was combined with the eluate from the previous step and diluted with 0.4 mL of DIL. mix. Next, M-280 Streptavidin Dynabeads (prewashed twice with 1 mL of WASH buffer and resuspended in 50 µL of DIL. mix) were added and the mixture was rotated for 30 min at room temperature. The beads were washed three times with 1 mL of WASH buffer, resuspended in 100 µL of TEN buffer (10 mM Tris-HCl at pH 8.0, 1 mM EDTA, 150 mM NaCl), and transferred to a new tube. The beads immobilized on the magnetic stand were rinsed with an additional 1 mL of TEN buffer. On-bead protein digestion followed exactly as described above in the nTAP-MS section.

### Mass spectrometry analysis

Digested peptides were acidified with 0.8% TFA (final) and analyzed by LC–MS/MS on an EASY-nLC 1000 (Thermo Scientific) with a two-column setup. The nTAP-MS peptides were applied on an Acclaim PepMap 100 C18 trap column (75 µm ID × 2 cm, 3 µm; Thermo Scientific) in 0.1% formic acid and 2% acetonitrile in H_2_O at a constant pressure of 80 MPa and separated by a linear gradient of 2%–6% buffer B in buffer A for 3 min, 6%–22% for 40 min, 22%–28% for 9 min, 28%–36% for 8 min, and 36%–80% for 1 min, and 80% buffer B in buffer A for 14 min (buffer A: 0.1% formic acid; buffer B: 0.1% formic acid in acetonitrile) on an EASY-Spray column ES801 (50 µm ID × 15 cm, 2 µm; Thermo Scientific) mounted on a DPV ion source (New Objective) connected to an Orbitrap Fusion (Thermo Scientific) at 150 nL/min flow rate. The xTAP-MS peptides were applied on a µPAC trapping column (PharmaFluidics) in 0.1% formic acid and 2% acetonitrile in H_2_O at a constant flow rate of 5 µL/min and separated by a linear gradient of 3%–6% buffer B in buffer A for 4 min, 6%–22% for 55 min, 22%–40% for 4 min, and 40%–80% for 1 min, and 80% buffer B in buffer A for 10 min on a 50-cm µPAC column (PharmaFluidics), mounted on an EASY-Spray source (Thermo Scientific) connected to an Orbitrap Fusion Lumos (Thermo Scientific) at 500 nL/min flow rate. Data were acquired using 120,000 resolution for the peptide measurements in the Orbitrap and a top T (3-sec) method with HCD fragmentation for each precursor and fragment measurement in the ion trap following the manufacturer guidelines (Thermo Scientific).

Peptides were identified with MaxQuant version 1.5.3.8 using the search engine Andromeda (Cox et al. 2011). The mouse subset of the UniProt version 2019_04 combined with the contaminant DB from MaxQuant was searched and the protein and peptide FDR values were set to 0.05. Statistical analysis was done in Perseus version 1.5.2.6 (Tyanova et al. 2016). Results were filtered to remove reverse hits, contaminants and peptides found in only one sample. Missing values were imputed and potential interactors visualized in the volcano plots were determined using a two-sided *t*-test.

### Coimmunoprecipitation with Streptavidin

Cells were seeded 3 × 10^5^ in 2 mL of S-L-2i medium per well of a six-well plate and transfected with a mixture of 200 ng of plasmid DNA, 0.6 µL of Lipofectamine 3000, and 0.4 µL of P3000 reagent in 100 µL of Opti-MEM medium. After 24 h, cells were rinsed twice with ice-cold PBS and lysed directly in the well with 900 µL of co-IP buffer (20 mM Tris-HCl at pH 7.5, 150 mM NaCl, 1 mM EDTA, 1% Triton X-100) supplemented with 1× HALT and, where indicated, also with 1 µL of 10 mg/mL RNase A (Roche). Following 1-h agitation at 4°C, the lysates were collected and centrifuged at 16,000*g* for 5 min at 4°C. An amount of 2 µL of the cleared supernatants was transferred to a separate tube as the input sample and the rest was mixed with 100 µL of co-IP buffer containing M-280 Streptavidin Dynabeads. Prior to use, the beads (20 µL of the original slurry) were preblocked for 1 h at room temperature in PBS containing 0.5% cold water fish gelatin (Sigma) and 0.02% Tween-20, and washed twice with co-IP buffer. Following 1-h incubation at 4°C, the beads were separated from the lysate and washed four times with 1 mL of co-IP buffer before resuspending in 15 µL of 1× loading buffer (4× NuPAGE LDS sample buffer and 10× NuPAGE sample reducing agent [Thermo Fisher Scientific] diluted to 1× with H_2_O). An amount of 13 µL of 1× loading buffer was also added to the input samples. All samples were heated for 3 min at 95°C and subjected to western blotting as described in the Western blotting section below.

### Coimmunoprecipitation with anti-m3G antibody

Cells were harvested by trypsinization and counted. For each sample, one million cells were rinsed once with PBS and collected by centrifugation at 200*g* for 3 min. Cell pellets were resuspended in 1 mL of co-IP buffer supplemented with 1× HALT and incubated for 10 min on ice. The lysates were centrifuged at 16,000*g* for 5 min at 4°C. An amount of 10 µL of the supernatant was transferred to a separate tube as the input sample and stored at 4°C for the duration of the IP. The remaining supernatant was split in two halves, supplemented with 2 µL of anti-2,2,7-trimethylguanosine (anti-m3G) antibody (Sigma) and 1 µL of either 40 U/µL RNasin RNase inhibitor (Promega) or 10 mg/mL RNase A (Roche), and incubated overnight at 4°C with mixing. The next day, 50 µL of Protein G Dynabeads (equivalent to 20 µL of the original slurry, prewashed twice with 1 mL of co-IP buffer) resuspended in co-IP buffer with 1× HALT was added and incubated for 4 h at 4°C with mixing. Beads were then washed three times with 1 mL of cold co-IP buffer before resuspending in 15 µL of 1× loading buffer. The inputs were mixed with 4 µL of 4× NuPAGE LDS sample buffer and 1.6 µL of 10× NuPAGE sample reducing agent. All samples were heated for 3 min at 95°C and subjected to western blotting as described in the Western blotting section below.

### Western blotting

Cells were harvested by trypsinization and counted. One million cells were rinsed once with PBS and collected by centrifugation at 200*g* for 3 min. Cell pellets were resuspended in 100 µL of TMNS buffer (10 mM Tris-HCl at pH 8.0, 1 mM MgCl_2_, 100 mM NaCl, 1% SDS) containing 25 U of Benzonase and incubated for 5 min at room temperature. An amount of 10 µL of the resulting lysate was mixed with 4 µL of 4× NuPAGE LDS sample buffer and 1.6 µL of 10× NuPAGE sample reducing agent, incubated for 3 min at 95°C and loaded on NuPAGE 4%–12% Bis-Tris Mini Protein Gel (Thermo Fisher Scientific). Proteins were separated by electrophoresis in NuPAGE MOPS SDS running buffer and transferred in Bjerrum and Schafer-Nielsen transfer buffer (48 mM Tris, 39 mM glycine, 20% methanol) onto Immobilon-P PVDF membrane (Millipore), using Trans-Blot SD Semi-Dry Transfer Cell (Bio-Rad) at a constant 15 V for 15 min, followed by a constant 25 V for 25 min. We note that for full-length NRDE2, using the rapid Trans-Blot Turbo Transfer System (Bio-Rad) resulted in suboptimal transfer that cannot be explained by the protein size. For visualization of biotinylated proteins, the membrane was blocked for 15 min at room temperature in TBST containing 1% BSA before adding HRP-Streptavidin (Sigma) to 1:20,000 dilution and incubating for a further 30 min. The membrane was washed three times (5 min each) with TBST, rinsed with PBS, and the proteins were visualized with Immobilon Western Chemiluminescent HRP substrate (Merck/Millipore; used at 1:1:3 [H_2_O] dilution) using Amersham Imager 600 (GE Healthcare). For antibody-based western blotting, the membrane was blocked for 10 min at room temperature in TBST containing 1% nonfat dry milk (TBST-NFDM) and then incubated with primary antibody diluted in TBST-NFDM with 0.05% NaN_3_ overnight at 4°C. The membrane was washed three times (5 min each) with TBST and incubated for 1 h at room temperature with HRP-conjugated secondary antibody diluted 1:10,000 in TBST-NFDM. After that, the membrane was washed, and the signal was developed as described above. When reprobing the same membrane with a different antibody, the membrane was stripped twice for 5 min in 25 mM glycine (pH 2.0), with the first incubation supplemented with 1% SDS, before rinsing thoroughly with water and repeating the blocking and antibody incubations as described above.

### Yeast two-hybrid screen

The Y2H screen was performed as a commercial service by Hybrigenics S.A. A bait cDNA of full-length mouse *Nrde2* (encoding amino acids 2–1172) was probed against Mouse Embryonic Stem Cell_RP1 cDNA library with a total of 58.2 million interactions tested. Gene Ontology terms of 87 high-confidence interactors (PBS score A–D) were analyzed using the clusterProfiler R package (Yu et al. 2012).

### Live-cell imaging

Ibidi µ-Slide 8-well chambers were coated with 10 µg/mL Biolaminin LN-511 (BioLamina) diluted in PBS with 1 mM CaCl_2_ and 0.5 mM MgCl_2_ overnight at 4°C. Coated wells were rinsed with PBS and immediately filled with S-L-2i medium to which 4 × 10^4^ to 6 × 10^4^ cells were plated. Cells were imaged 24 h after seeding on a Nikon Ti2-E Eclipse inverted microscope equipped with a Yokogawa CSU W1 spinning disk confocal scanning unit, two back illuminated EMCCD iXon-Ultra-888 (Andor) cameras and CFI Plan Apochromat Lambda 100×/1.45 oil immersion objective (Nikon). Fluorescence was excited with 488-nm iBeam Smart (Toptica) and 561-nm Cobolt Jive (Cobolt) lasers, and images (pixel size 0.13 µm) were acquired using VisiView software (Visitron Systems GmbH) with the following settings: 100% laser intensity, 500-msec exposure time, EMCCD GAIN 100 for all mNeonGreen-tagged proteins and 25% laser intensity, 200-msec exposure time, EMCCD GAIN 100 for mCherry-U2AF2. The cells were kept at 37°C and 5% CO_2_ during all treatments and imaging.

### BCLIP-seq of 3A-tagged proteins

For each BCLIP-seq sample, 1.5 × 10^7^ cells were seeded on a 10-cm dish 24 h before cross-linking. For low-abundant proteins, two or four dishes were seeded per sample and processed separately until pooling two at a time during the last FLAG beads wash and, when starting with four dishes, during the last Streptavidin beads wash as described below. Cells were rinsed once on ice and then covered with 5 mL of cold PBS. Open dishes without a lid were placed on an ice-cold aluminum plate in Stratalinker 2400 (Stratagene), and 254 nm UV light was applied for 30 sec to cross-link the protein–RNA interactions. After cross-linking, PBS was removed, cells were scraped in 2 × 0.9 mL of 0.2% BSA in cold PBS and pelleted by spinning at 3000*g* for 2 min at 4°C. Pellets were resuspended by vortexing in 100 µL of TMS buffer (as in xTAP-MS) prechilled at 12°C and supplemented with 100 U of Benzonase. Following 30-min incubation at 12°C, the lysates were diluted with 0.85 mL of DIL. mix (as in xTAP-MS) and incubated for 5 min on ice. Any remaining insoluble material was removed by spinning at 16,000*g* for 5 min at 4°C.

The cleared lysates were mixed with anti-FLAG antibody-coupled Protein G Dynabeads resuspended in 50 µL of DIL. mix (as in xTAP-MS) and incubated for 2 h rotating at 4°C, followed by washes, 3xFLAG peptide elution, and incubation with M-280 Streptavidin Dynabeads exactly as described above for xTAP-MS. The Streptavidin beads were washed three times with 1 mL of WASH buffer, once with 0.2 mL of 1× PAP buffer (50 mM Tris-HCl at pH 8.0, 250 mM NaCl, 10 mM MgCl_2_, 0.02% Tween-20) and transferred in 0.1 mL of 1× PAP to a 0.2-mL PCR tube.

The purified cross-linked RNA fragments were polyadenylated by resuspending the beads in 20 µL of 1× PAP buffer containing 0.1 mM ATP and 2.5 U *Escherichia coli* Poly(A) polymerase (NEB), and incubating for 10 min at 37°C with interval mixing (2000 RPM for 15 sec every 3 min). The reaction was stopped by adding 180 µL of TES buffer (10 mM Tris-HCl at pH 8.0, 1 mM EDTA, 2% SDS) and the beads were washed two more times with 0.2 mL of TES before resuspending in 20 µL of TET buffer (10 mM Tris-HCl at pH 8.0, 1 mM EDTA, 0.5% Triton X-100) with 0.5 µL of 20 mg/mL Proteinase K (Roche). After 30 min of protein digestion at 50°C with interval mixing, Proteinase K was inactivated for 3 min at 85°C. The beads were separated on a magnet, and the supernatant was transferred to a new tube and mixed with 20 µL of homemade SPRI beads (equivalent of 1 mL of Sera-Mag Magnetic SpeedBeads, carboxylated, 1-µm 3 EDAC/PA5 [GE Healthcare Life Sciences] in 50 mL of binding buffer containing 10 mM Tris-HCl at pH 8.0, 1 mM EDTA, 2.5 M NaCl, 20% PEG8000, 0.05% Tween-20, and 0.05% NaN_3_) and 40 µL of isopropanol. RNA was precipitated rotating for 10 min at room temperature, beads were separated and rinsed on a magnet twice with 0.2 mL of 80% ethanol and dried for 1 min, and finally RNA was eluted in 15.25 µL of 0.02% Tween-20.

The purified RNA was mixed with 1 µL of 0.2 µM RA3dT18V primer and 1 µL of 10 mM dNTPs (10 mM each dATP, dCTP, dGTP, dTTP), denatured for 3 min at 65°C, and quickly cooled on ice. Reverse transcription was started by mixing with a master mix consisting of 2 µL of 10× RT buffer (500 mM Tris-HCl at pH 8.3, 750 mM KCl, 30 mM MgCl_2_, 20 mM TCEP), 0.5 µL of RNase Block (Agilent), and 0.25 µL of SuperScript II (Thermo Fisher Scientific) and incubating for 15 min at 42°C, after which 1 µL of 40 mM MnCl_2_ and 1µL of 20 µM TSO were added, and the 42°C incubation continued for another 30 min followed by a 15-min heat inactivation at 70°C. The reaction was slowly cooled down to room temperature with a Δ −1°C/sec gradient, and 0.5 µL each of 5 U/µL RNase H (NEB), 1000 U/µL RNase T1 (Thermo Fisher Scientific), and 20 U/µL Exonuclease I (NEB) were added to degrade the excess TSO and RT primer for 20 min at 37°C, followed by a 15-min heat inactivation at 80°C. The resulting cDNA was purified by isopropanol precipitation on SPRI beads as described above and eluted in 17.2 µL of 0.02% Tween-20.

To amplify the cDNA library, the eluates from the previous step were transferred to a 0.2-mL low-profile PCR tube (Bio-Rad) and mixed with a master mix consisting of 20 µL of 2× NEBNext Ultra II Q5 Master Mix (NEB), 1 µL of 40 µM P5* primer, 1 µL of 40 µM PE* primer, and 0.8 µL of 100 µM EvaGreen Fluorescent DNA Stain (Jena Bioscience). The reactions were amplified in a CFX96 Real-Time PCR instrument (Bio-Rad) with an initial denaturation for 45 sec at 98°C, followed by cycles of 10 sec at 98°C and 50 sec at 65°C, plate read, and10 sec at 65°C. The amplification was monitored in real time, and the reactions were removed after three cycles of exponential fluorescence signal increase (typically between 12 and 18 total cycles). The amplified library was purified by mixing with 32 µL of homemade SPRI beads and incubating for 5 min at room temperature. The beads were then separated and rinsed on a magnet twice with 0.2 mL of 80% ethanol and dried for 1 min, and DNA was eluted in 20 µL of H_2_O. Equal amounts of libraries generated with different barcode TSOs were pooled together and sequenced on the Illumina HiSeq2500 platform (50-nt single-end reads).

All BCLIP-seq related oligonucleotides were ordered as standard desalted RNA/DNA oligos or ultramers from IDT and their sequences are listed in Supplemental Table S3.

### RNA-seq

Total RNA was isolated from near-confluent 6-cm dishes using the Absolutely RNA Miniprep kit (Agilent), including an on-column DNase treatment step, according to the manufacturer's instructions. Libraries were prepared using the TruSeq Stranded Total RNA Library Prep Human/Mouse/Rat kit (Illumina), including a ribosomal RNA depletion step, and sequenced on the Illumina HiSeq2500 platform (50-nt single-end reads).

### Quantitative RT-PCR

Total RNA was isolated as described in the RNA-seq section and 500 ng was reverse-transcribed using the PrimeScript RT Master Mix (Takara). A volume of cDNA corresponding to 5 ng of the input RNA was subjected to quantitative PCR (qPCR) in a 10-µL reaction using the SsoAdvanced Universal SYBR Green Supermix (Bio-Rad) and 0.4 µM each forward and reverse primer. The qPCR was performed in a CFX96 Real-Time PCR instrument (Bio-Rad) with an initial denaturation for 30 sec at 95°C, followed by 40 cycles of 5 sec at 95°C,10 sec at 60°C, and plate read. The relative RNA levels were determined using the ΔΔ*C*_t_ method and normalized to *Actb* expression. All qPCR primer sequences are listed in Supplemental Table S4.

### RT-PCR of splicing isoforms

For the splicing analysis of endogenous transcripts, cells were grown to near confluency on 6-cm dishes and total RNA was extracted using the RNAzol RT reagent (Sigma) following the manufacturer's protocol. An amount of 1 µg of the RNA was reverse-transcribed in a 10-µL reaction using SuperScript IV (Thermo Fisher Scientific) and random primers (Agilent), and a volume of cDNA corresponding to 5 ng of the input RNA was subjected to 30 cycles of PCR with the 2× NEBNext Ultra II Q5 Master Mix (NEB).

To determine the splicing pattern of the luciferase reporter transcripts, 2 × 10^5^ wild-type cells or 4 × 10^5^ *Nrde2*-KO cells (2 d of 4OHT treatment) were seeded on a six-well plate, transfected with 500 ng of the corresponding reporter plasmid DNA using Lipofectamine 3000, and grown for 48 h. Cells were harvested in 0.5 mL of RNAzol RT reagent supplemented with 1 µL of Polyacryl Carrier (MRC), and total RNA was extracted following the manufacturer's protocol. An amount of 500 ng of the RNA was treated with ezDNase (Thermo Fisher Scientific) and reverse transcribed using SuperScript IV and random primers. A volume of cDNA corresponding to 2.5 ng of the input RNA was subjected to 35 cycles of PCR with the 2× NEBNext Ultra II Q5 Master Mix (NEB).

The PCR reactions were purified using homemade SPRI beads (as described in the BCLIP-seq section), resolved on a 1% agarose gel containing Midori Green Advance stain (Nippon Genetics) and visualized using the FastGene FAS-DIGI PRO gel imaging system (Nippon Genetics). The sequences of primers used for splicing pattern analysis are listed in Supplemental Table S4.

### Ribo-seq

For each Ribo-seq sample, 2 × 10^6^ cells (3 d of 4OHT treatment for *Nrde2*-KO cells) were seeded on a 6-cm dish and grown overnight. Cells were rinsed and scraped in 2 × 0.5 mL of cold PBS on ice and pelleted by spinning at 3300*g* for 2 min at 4°C. The pellet was resuspended by pipetting in 0.2 mL of Ribo-seq buffer (25 mM HEPES-KOH at pH 7.5, 200 mM KOAc, 2 mM MgCl_2_, 1% NP-40) and incubated for 5 min on ice, followed by spinning at 7600*g* for 5 min at 4°C. An amount of 100 µL of the supernatant was transferred to a new tube, mixed with 100 µL of H_2_O and 0.4 µL of 250 U/µL Benzonase, and incubated for 30 min at 37°C with constant shaking. The nuclease digestion was stopped by adding 0.5 mL of RNAzol RT reagent with 1 µL of Polyacryl Carrier, and RNA fragments were purified following the manufacturer's total RNA isolation protocol. The precipitated RNA was resuspended in 5 µL of H_2_O, mixed with 5 µL of 2× Novex TBE-Urea sample buffer (Thermo Fisher Scientific) and denatured for 3 min at 70°C. RNA was resolved on a Novex 15% TBE-Urea gel (Thermo Fisher Scientific) in 1× TBE buffer for 75 min at 180 V side by side with 20 ng of a 35-nt-long single-stranded DNA oligo 5′-ACCACTCGAGTCAAAACAGAGATGTGTCGAAGATG-3′ (IDT). The gel was stained in 20 mL of 1× TBE buffer containing 2 µL of SYBR Gold stain (Thermo Fisher Scientific) for 5 min at room temperature, and a band migrating just above the 35-nt oligo was cut from the Ribo-seq sample lane.

The gel piece was submerged in 100 µL of 400 mM NaOAc (pH 5.2) and frozen at −80°C. After thawing the gel-containing tube for 5 min at 95°C, the gel piece was crushed with a plastic pestle, 300 µL of 400 mM NaOAc (pH 5.2) was added, and RNA was extracted from the gel by three cycles of 5-min incubation at 95°C and 20-min shaking at 22°C. The mixture was cleared by spinning through a Corning Costar Spin-X filter (0.45-µm cellulose acetate; Sigma) at 20,000*g* for 5 min at room temperature. RNA was precipitated by adding 1 µL of glycogen (Roche) and 1 mL of ethanol to the flow-through and incubating for 1 h at −20°C. RNA was pelleted by spinning at 16,000*g* for 15 min at 4°C, and the pellet was washed twice with 0.5 mL of cold 80% ethanol, dried for 5 min at room temperature, and finally resuspended in 10 µL of C1 buffer (10 mM Tris-HCl at pH 7.5, 1 mM EDTA, 1 M NaCl, 0.02% Tween-20).

Before the next step, custom Ribo-beads were prepared as follows. Streptavidin MyOne C1 Dynabeads (Thermo Fisher Scientific) equivalent to 20 µL of the original slurry were washed twice with 0.2 mL of C1 buffer and resuspended in 20 µL of C1 buffer. Two rRNA depletion oligos were ordered from IDT as 3′-biotinylated standard desalted custom DNA oligos CCGTACGCCACATTTCCCACGCCGCGACGCGC/3BioTEG/ and CAAGACGAACGGCTCTCCGCACCGGACCCCGGTCCC/3BioTEG/, resuspended, and mixed to 50 µM each. Before the first use, the mixture was denatured for 1 min at 95°C and quickly cooled on ice. An amount of 2 µL of the rRNA depletion oligo mix was added to the 20 µL of C1 Dynabeads and incubated for 15 min at room temperature with occasional mixing. The beads were washed twice with 0.2 mL of C1 buffer and resuspended in 20 µL of C1 buffer.

The purified RNA fragments were mixed with 10 µL of the Ribo-beads and incubated for 3 min at 70°C, followed by 10 min at 50°C. Beads were separated on a magnet, and the supernatant was mixed with the remaining 10 µL of the Ribo-beads. After another round of 3 min at 70°C and 10 min at 50°C, the supernatant was mixed with 30 µL of homemade SPRI beads (as in BCLIP-seq) and 50 µL of isopropanol, and the RNA was precipitated rotating for 10 min at room temperature. The beads were separated, rinsed on a magnet twice with 0.2 mL of 80% ethanol, and dried for 1 min, and finally RNA was eluted in 13.5 µL of 0.02% Tween-20.

The purified rRNA-depleted RNA fragments were polyadenylated by adding 4 µL of 5× PAP buffer (as in BCLIP-seq), 2 µL of 1 mM ATP, and 0.5 µL of 5 U/µL *E. coli* Poly(A) polymerase (NEB) and incubated for 10 min at 37°C with interval mixing (2000 RPM for 15 sec every 3 min). The reaction was stopped by the addition of 20 µL of homemade SPRI beads and 40 µL of isopropanol. Subsequent polyadenylated RNA purification, reverse transcription with template switching, cDNA library amplification, and sequencing on Illumina HiSeq2500 were performed exactly as described in the BCLIP-seq section.

### Flow cytometry

Cells from a near-confluent six-well plate were harvested by trypsinization and pelleted by spinning at 300*g* for 2 min. The pellets were washed once and resuspended in cold PBS. The mNeonGreen fluorescence of the endogenously expressed 2C reporter was measured on a BD LSR II flow cytometer (BD Biosciences) using a 488 nm excitation laser and a FITC filter. The acquired data were analyzed using the FlowJo software.

### Luciferase reporter assays

The backbone control luciferase reporter plasmid was created by placing the Renilla and Firefly luciferase coding sequences with SV40 and BGH polyA sites, respectively, under the control of a minimal endogenous bidirectional promoter driving the expression of mouse *Emg1* and *Phb2* genes, into the pBluescript II KS-plasmid (Agilent Technologies). For in-frame fusion with Cdk2 and Tti1 fragments, a P2A self-cleaving peptide sequence was included to separate the Renilla luciferase. Site-directed mutagenesis was performed by PCR with NEBNext High-Fidelity 2× PCR master mix (NEB) using overlapping primers with 15-nt overhangs carrying the desired mutation (IDT).

Cells were seeded 2 × 10^5^ to 6 × 10^5^ per well of a 96-well plate and transfected with 10 ng of the corresponding reporter plasmid supplemented with 90 ng of pBluescript II KS, using Lipofectamine 3000. *Nrde2*-KO cells were treated with 4OHT for 2 d prior to transfection. Where appropriate, the cells were treated with the dTAG-13 compound 24 h post-transfection. After 48 h post-transfection, cells were washed with PBS and lysed in 25 µL of Passive Lysis Buffer (Promega) for 20 min at room temperature. An amount of 10 µL of the lysate was used to measure luciferase activity by sequential mixing with 10 µL of each substrate from the Dual-Luciferase Reporter Assay System (Promega). Luminescence was measured on a Mithras LB 940 microplate reader (Berthold Technologies). All Renilla luminescence values were first normalized to Firefly and the normalized activity was then calculated relative to the empty control reporter in wild-type cells.

### RNA immunoprecipitation followed by northern blotting

Cells were harvested by trypsinization and counted. For each sample, 5 × 10^7^ cells were collected by centrifugation at 300*g* for 2 min and rinsed once with 1 mL of PBS. Cell pellets were resuspended in 1 mL of cold co-IP buffer (20 mM Tris-HCl at pH 7.5, 150 mM NaCl, 1 mM EDTA, 1% Triton X-100) supplemented with 1× HALT in a 15-mL Bioruptor tube containing one scoop of sonication beads (Diagenode) and sonicated in Bioruptor Pico (Diagenode) in five cycles of 30 sec ON/30 sec OFF. Lysates were cleared by centrifugation at 16,000*g* for 5 min at 4°C, and 1 µL of the supernatant was taken to a separate tube as 0.1% input. The remaining supernatant was mixed with anti-FLAG antibody-coupled Protein G Dynabeads (prepared as described above in the nTAP-MS section, but prewashed with 2 × 1 mL of co-IP buffer and resuspended in 50 µL of co-IP buffer with 1× HALT). After 2 h rotating at 4°C, the beads were washed three times with 1 mL of cold co-IP buffer, and FLAG-tagged proteins were eluted by two consecutive 15-min incubations with shaking (500 RPM) at room temperature—first in 100 µL of co-IP buffer with 1× HALT containing 0.25 mg/mL 3xFLAG peptide, and second in 25 µL of co-IP buffer with 1× HALT containing 1 mg/mL 3xFLAG peptide. The beads were rinsed with another 325 µL of co-IP buffer, and the supernatant was combined with the eluates from the previous step. M-280 Streptavidin dynabeads (prewashed twice with 1 mL of co-IP buffer) resuspended in 50 µL of co-IP buffer with 1× HALT were then added, and the samples were incubated for 30 min at room temperature with mixing. Beads were washed on ice three times with 1 mL of cold co-IP buffer and resuspended in 0.5 mL of RNA extraction buffer (10 mM Tris-HCl at pH 8.0, 10 mM EDTA, 1% SDS, 350 mM NaCl, 7 M Urea), which was also added to the inputs. RNA was extracted by adding 0.5 mL of buffered phenol:chloroform:IAA (pH 8.0; Sigma) and shaken for 1 min, followed by spinning at 20,000*g* for 5 min at room temperature. The upper phase was transferred to a new tube, and RNA was precipitated by mixing with an equal volume of isopropanol in the presence of 1 µL of Polyacryl Carrier (MRC). After 10-min incubation at room temperature, RNA was pelleted by centrifugation at 16,000*g* for 30 min at 4°C, washed once with 0.5 mL of 75% ethanol, air-dried for 5 min, and resuspended in 5 µL of water. Next, 5 µL of 2× Novex TBE-Urea Sample Buffer (Thermo Fisher Scientific) was added, and samples were denatured for 3 min at 95°C and immediately cooled on ice. RNA was separated on a 6% Novex TBE-Urea gel (Thermo Fisher Scientific) for 70 min at 90 V in 1× TBE buffer preheated to 50°C. The gel was briefly rinsed with 1× TBE, and RNA was transferred to positively charged Nylon membrane (Roche) using Fastblot (Biometra) for 30 min at a constant current of 200 mA. After the transfer, RNA was cross-linked to the wet membrane for 90 sec using Stratalinker 2400. The membrane was air-dried, washed in 2× SSC buffer containing 0.1% SDS, and prehybridized in 10 mL of ULTRAhyb-Oligo buffer (Thermo Fisher Scientific) for 90 min at 37°C. Twenty picomoles of a 3′-end biotinylated ssDNA oligo (GTATCTCCCCTGCCAGGTAAGTAT/Bio/ for U1 snRNA and TACTGCAATACCAGGTCGATGCGT/Bio/ for U2 snRNA) (Ishikawa et al. 2014) was then added and hybridized overnight at 37°C. The membrane was washed three times for 15 min with 50 mL of prewarmed 2× SSC buffer containing 0.5% SDS at 37°C, and the biotin signal was detected with a Chemiluminescent Nucleic Acid Detection Module kit (Thermo Fisher Scientific) following the manufacturer's instructions. The signal was visualized using Amersham Imager 600 (GE Healthcare).

### Quantification and statistical analysis

#### Live-cell imaging signal quantification

All microscopy image signal quantification was performed in Fiji (Schindelin et al. 2012). NRDE2 and U2AF2 intensity profiles in Supplemental Figure S3A,C (supporting Fig. 1G–I) were measured using the standard inbuilt profile plotting algorithm within straight, manually drawn 1-pixel width lines. The intensity values were extracted and normalized to the maximal intensity measured within each profile–protein pair, before plotting using Prism 8.3.0. Mean intensities of NRDE2 and CCDC174 in Figure 5D (supporting Fig. 5C) were measured within masks generated according to manually adjusted intensity thresholds based on the U2AF2 signal to encompass the entire nucleus. Background measurements were taken from manually drawn boxes outside any cells and subtracted from the NRDE2 and CCDC174 values, before plotting and statistical analysis using Prism 8.3.0.

#### BCLIP-seq data preprocessing and alignment

BCLIP-seq reads were preprocessed as described previously for the CRAC method (Tuck et al. 2020). Briefly, adapters and low-quality bases were trimmed and duplicate reads were collapsed. Samples were split according to their barcodes, and low-complexity regions were removed from the 3′ ends of the reads.

The preprocessed reads were aligned (splicing-aware) to the mouse genome (mm10), including the transcriptome annotation of GENCODE release M23 using STAR version 2.7.0a (Dobin et al. 2013), with the parameters –outFilterMultimapNmax 20 and –outFilterMismatchNoverLmax 0.05.

For BCLIP-seq read categorization (Fig. 2B), noncollapsed reads were aligned to the mouse ribosomal DNA repeating unit (GenBank: BK000964) using Bowtie2 version 2.3.5.1 (Langmead and Salzberg 2012) with –sensitive parameters. Then the unmapped reads were mapped in the same way to GENCODE release M23 protein-coding transcripts. Finally, the leftover unmapped reads were mapped with the same parameters to the mm10 mouse genome. Mapped reads were counted using SAMtools flagstat version 1.10 (Li et al. 2009).

To analyze snRNA-mapping reads, noncollapsed reads were aligned to snRNA sequences using Bowtie2 version 2.3.5.1 with –sensitive parameters. Reads that were at least 20 bp long and mapped with less than 1 edit distance (NM:i:0 or NM:i:1) were used to calculate coverage across snRNAs.

#### RNA-seq read alignment

RNA-seq reads were aligned (splicing-aware) to the mouse genome (mm10), including the transcriptome annotation of GENCODE release M23 using STAR version 2.7.3a with the parameters –outFilterMultimapNmax 100, –outFilterMismatchNoverLmax 0.05, and –outSAMmultNmax 1.

#### Selection of expressed 5′ splice sites

GENCODE release M23 annotation was used for all analysis unless otherwise stated. The number of reads in all genes for all WT samples were counted using featureCounts from the Rsubread package (Liao et al. 2019) with the following parameters: allowMultiOverlap = FALSE, minOverlap = 1, countMultiMappingReads = FALSE, fraction = FALSE, minMQS = 255, and strandSpecific = 2.

All genes with FPKM > 0 were used as expressed genes. In addition, transcript abundance was quantified using Salmon version 1.2.0 (Patro et al. 2017) with the parameters -l A -r –validateMappings –gcBias, and only transcripts with TPM > 0 were kept. Then introns (and splice sites) were extracted from these transcripts using the GenomicFeatures R package (Lawrence et al. 2013) and annotated using the EnsDb.Mmusculus.v79 R package.

Heat maps were generated using the MiniChip R package (https://github.com/fmi-basel/gbuehler-MiniChip): Coverage was calculated in regions ±200 bp around all 5′SSs, using only uniquely mapping reads to the forward strand, and displayed as the average cpm across two replicates.

### Analysis of 5′ splice site-overlapping BCLIP-seq reads

The regions ±100 bp around 5′SSs of introns >1 kb were selected. Those with less than 50 BCLIP-seq reads (in total over all samples) were removed. Each BCLIP-seq .bam file was loaded using RSamtools R package, and the reads that overlap 5′SSs were selected. Spliced or unspliced reads were counted separately for each junction and each sample .bam file. Then the 5′SSs with spliced and unspliced read counts above 10 were selected, and the fold change between spliced and unspliced read counts was calculated.

#### Differential gene expression analysis

The number of correctly stranded uniquely mapping RNA-seq reads was quantified in genes using featureCounts (Rsubread package). CHX-treated and untreated samples were analyzed separately using DEseq2 (Love et al. 2014), first filtering out genes that have less than 200 counts across all samples or 80 counts across all CHX samples. Genes with an adjusted *P*-value <0.01 and log_2_FC >1/−1 were counted as up-/down-regulated.

#### Differential repeat expression analysis

Repeat expression was quantified with featureCounts using RepeatMasker tracks for LTR, LINE, SINE, and DNA elements, removing repeats that overlap genes, using only uniquely mapping reads. The counts were normalized using the TMMwsl method from the edgeR package (Robinson et al. 2010), taking the total number of uniquely mapped reads as library size. Repeats with the maximum cpm below 0.5 were filtered out and the *limma* R package (Ritchie et al. 2015) was used to calculate differential expression. The average log_2_FC across all genomic instances of a given repeat element (with at least five genomic locations) was then calculated.

#### Differential intron expression analysis

The number of correctly stranded uniquely mapping RNA-seq reads was quantified in expressed introns using featureCounts with the parameters useMetaFeatures = FALSE, allowMultiOverlap = TRUE, minOverlap = 10, countMultiMappingReads = FALSE, fraction = FALSE, minMQS = 255, and strandSpecific = 2.

CHX-treated and untreated samples were analyzed separately using DEseq2, first filtering out introns that have less than 110 counts across all samples or 40 counts across all CHX samples. Introns with an adjusted *P*-value <0.05 and log_2_FC >0.5/−0.5 were counted as up/down-regulated. Finally, up-regulated introns within genes that are not up-regulated (log_2_FC < 0) were selected for further analysis.

#### Intron features and alternative splicing

All intron features (GC content, length, splicing scores) were calculated using Matt (Gohr and Irimia 2018). Fastq files of replicates within a group were combined and mapped to the genome using HISAT2 version 2.1.0 (Kim et al. 2019), followed by transcriptome reconstruction using StringTie version 2.1.1 (Pertea et al. 2015). All resulting .gtf files were merged and annotated using GffCompare version 0.11.6 (Pertea and Pertea 2020). All rMATS (Shen et al. 2014) comparisons were done using the resulting .gtf file. Retained introns, novel 5′SSs or 3′SSs were selected based on FDR < 0.01 and inclusion level difference greater than 0.1, including only introns with more than 30 reads across all samples.

#### Peak finding

CLIPper tool (Lovci et al. 2013) was used to find peaks in STAR-mapped collapsed low-complexity-stripped BCLIP-seq .bam files. Peaks that overlapped between the two replicates, were >35 bp, and contained more than five reads (in both replicates combined) in a 50-bp window around the peak center, were kept.

#### Ribo-seq analysis

Ribo-seq reads were preprocessed as described for BCLIP-seq. Read mapping and differential gene expression analysis for Ribo-seq data were performed as for RNA-seq data. Genes with less than 10 reads across all samples were excluded from differential expression analysis[Table RNA079465FLETB1].

## DATA DEPOSITION

All reagents and cell lines generated in this study are available from the corresponding author, Marc Bühler (marc.buehler@fmi.ch), upon a Materials Transfer Agreement. All sequencing data generated in this study have been deposited at NCBI GEO and are available under accession number GSE179744. Mass spectrometry data have been deposited at ProteomeXchange via the PRIDE database under accession number PXD029392. Original western and northern blot images have been deposited at Mendeley Data and can be accessed at https://data.mendeley.com/datasets/v24ytccs4h/2. Custom scripts and tools for data analysis are available on GitHub (https://github.com/xxxmichixxx/Nrde2Project) or are indicated in the respective Materials and Methods sections.

## SUPPLEMENTAL MATERIAL

Supplemental material is available for this article.

## COMPETING INTEREST STATEMENT

The Friedrich Miescher Institute for Biomedical Research (FMI) receives significant financial contributions from the Novartis Research Foundation. Published research reagents from the FMI are shared with the academic community under a Material Transfer Agreement (MTA) having terms and conditions corresponding to those of the UBMTA (Uniform Biological Material Transfer Agreement).

## Supplementary Material

Supplemental Material

## Figures and Tables

**Matyas Flemr:**
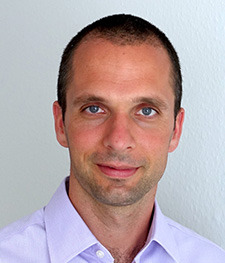


## MEET THE FIRST AUTHOR

**Meet the First Author(s) is an editorial feature within *RNA*, in which the first author(s) of research-based papers in each issue have the opportunity to introduce themselves and their work to readers of *RNA* and the RNA research community. Matyas Flemr is the first author of this paper “Mouse nuclear RNAi-defective 2 promotes splicing of weak 5′ splice sites.” During the time working on the project presented in this manuscript, Matyas was a post-doc in the laboratory of Professor Marc Bühler at Friedrich Miescher Institute in Basel, Switzerland. The main focus of his research was on pre-mRNA splicing regulation in mouse embryonic stem cells, additionally focusing on developing and optimizing techniques to decipher protein–protein and protein–RNA interactions. Currently, he is a Principal Scientist at Vector BioPharma AG in Basel, Switzerland, focusing on therapeutic CRISPR/Cas9-based gene editing and its delivery by adenovirus-based virus-like particles.**

**What are the major results described in your paper and how do they impact this branch of the field?**

Our results provide an insight into correct 5′ splice selection within introns with suboptimal (weak) 5′ splice site complementarity to U1 snRNA during pre-mRNA splicing in mammalian cells. The field of splicing regulation and splice site usage has been mostly focusing on proximal regulatory sequences within pre-mRNA; that is, splice enhancers and splice repressors, and the splicing regulatory factors that bind them, mainly from the SRSF and HNRNP protein families. We show that a conserved protein NRDE2, together with its interactor CCDC174, binds to U1 snRNA and acts directly at weak 5′ splice sites to promote their usage. Our results put a new perspective on the composition of spliceosome complexes with specialized functions.

**What led you to study RNA or this aspect of RNA science?**

While finishing my undergraduate studies in Biochemistry and Plant Enzymology in the mid 2000s, I became fully absorbed by the then ongoing RNAi wave and went on to pursue a PhD studying miRNAs and siRNAs in mouse oocytes. I wanted to capitalize on my small RNA expertise during my post-doc and chose to study the function of a conserved protein NRDE2 in mammalian cells. At that time, NRDE2 was shown to link endogenous siRNAs to chromatin regulation in worms and fission yeast. However, as it emerged over the years working on this project, mammalian NRDE2 is mostly involved in pre-mRNA splicing regulation. Hence, I became a self-taught splicing aficionado.

**During the course of these experiments, were there any surprising results or particular difficulties that altered your thinking and subsequent focus?**

This project has been a continuous string of surprising results, starting with the hypothesis that NRDE2 together with MTREX regulates retroelements via heterochromatin and ending with characterizing NRDE2 as an MTREX-independent weak 5′ splice site regulator. NRDE2 characterization came with a lot of technical struggles as it turned out to be a tricky protein to work with in vitro. This in turn led to a lot of troubleshooting and methods optimization, an area that I greatly enjoy. The troubleshooting and optimization training has equipped me perfectly for my current job working in a biotech start-up!

**If you were able to give one piece of advice to your younger self, what would that be?**

Don't be too stubborn and don't spend too much time on a tricky project beyond the scope of your or your lab's expertise. Though, in that case, this NRDE2 project would have never been brought anywhere close to the state in which it is now published.

**What are your subsequent near- or long-term career plans?**

I have now switched from academia to a biotech start-up environment, where I can fully focus on technology development, an area that I greatly enjoy. Academia and industry are often described as two completely different worlds. However, in the end it's all about the people you work with and in that regard I have been extremely lucky in both worlds.
