# Supplementary material for: Mouse nuclear RNAi-defective 2 promotes splicing of weak 5′ splice sites
Source: RNA. 2023 Aug;29(8):1140–65. doi: 10.1261/rna.079465.122 (PMC10351895; doi:10.1261/rna.079465.122)
Supplement: Supplemental Material [file supp_29_8_1140__DC1.html]

Mouse nuclear RNAi-defective 2 promotes splicing of weak 5′ splice sites — Supplemental Material 

# Mouse nuclear RNAi-defective 2 promotes splicing of weak 5′ splice sites

## Supplemental Material

- Supplemental\_FigS1.tif
- Supplemental\_FigS2.tif
- Supplemental\_FigS3.tif
- Supplemental\_FigS4.tif
- Supplemental\_FigS5.tif
- Supplemental\_FigS6.tif
- Supplemental\_FigS7.tif
- Supplemental\_FigS8.tif
- Supplemental\_Table\_S1.xlsx
- Supplemental\_Table\_S2.xlsx
- Supplemental\_Table\_S3.xlsx
- Supplemental\_Table\_S4.xlsx
- Supplemental\_Legends.docx
