# Supplementary figures and images for: Mouse nuclear RNAi-defective 2 promotes splicing of weak 5′ splice sites
Source: RNA. 2023 Aug;29(8):1140–65. doi: 10.1261/rna.079465.122 (PMC10351895; doi:10.1261/rna.079465.122)

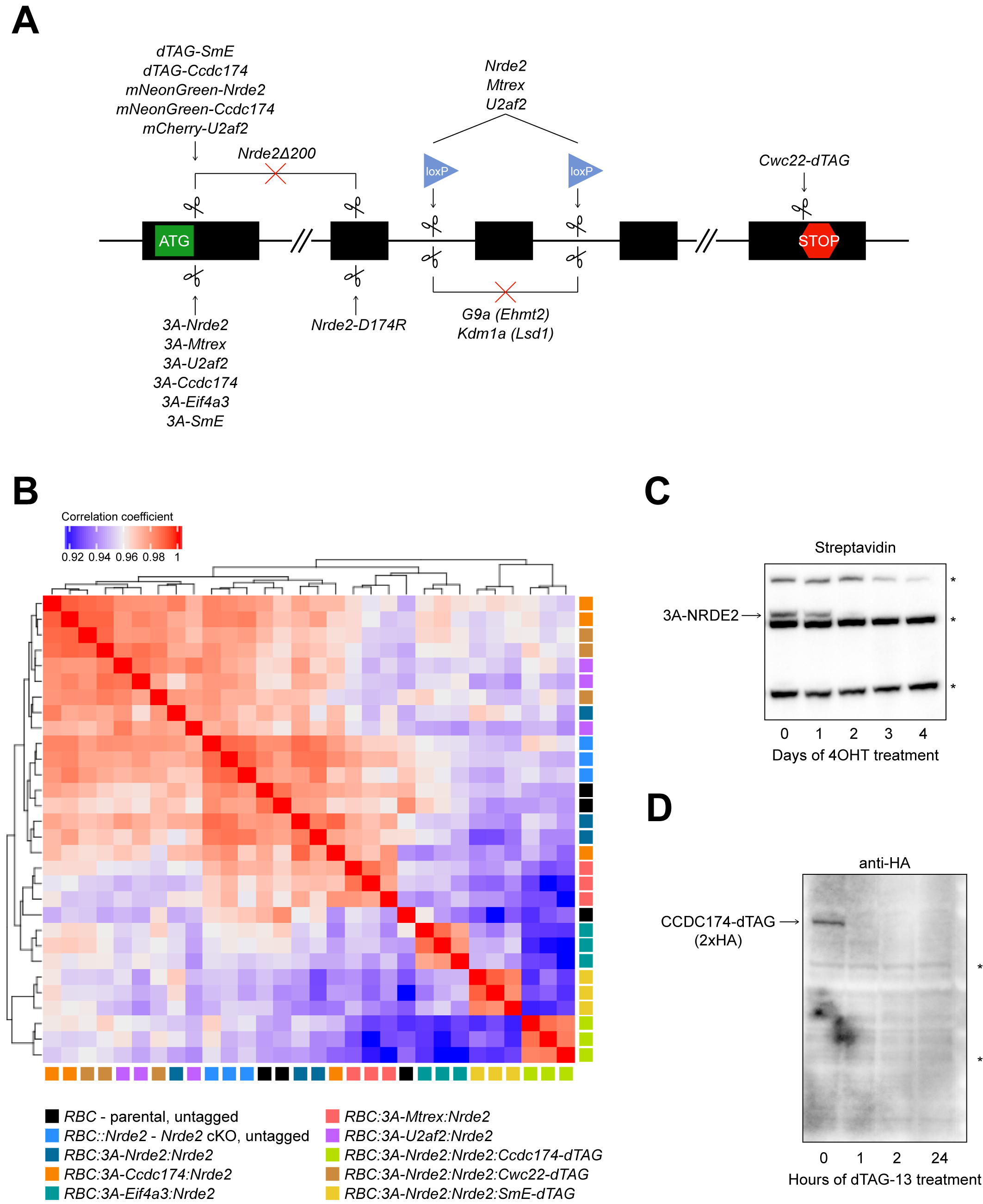

Supplement: Supplemental Material [file supp_079465.122_Supplemental_FigS1.tif]

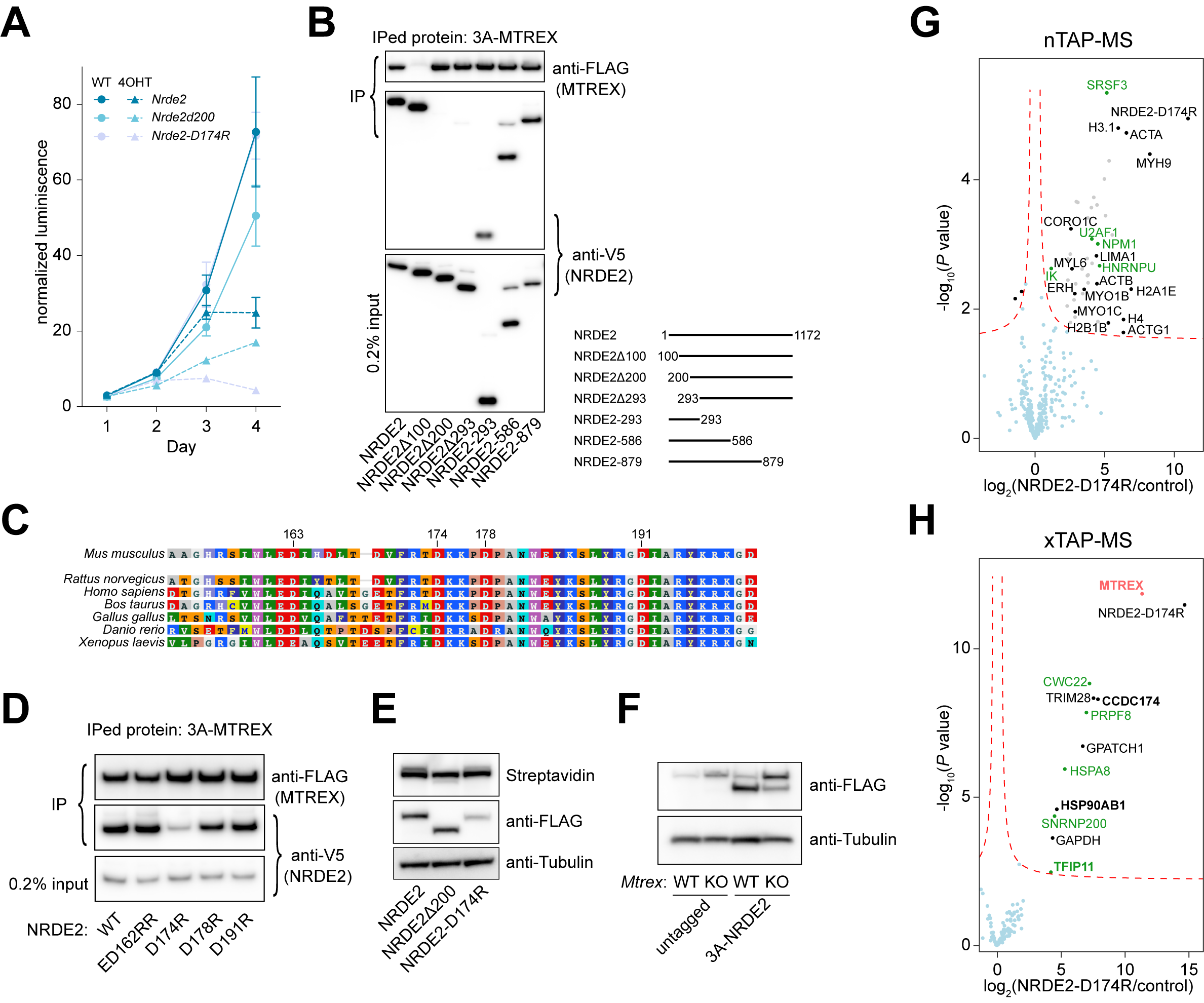

Supplement: Supplemental Material [file supp_079465.122_Supplemental_FigS2.tif]

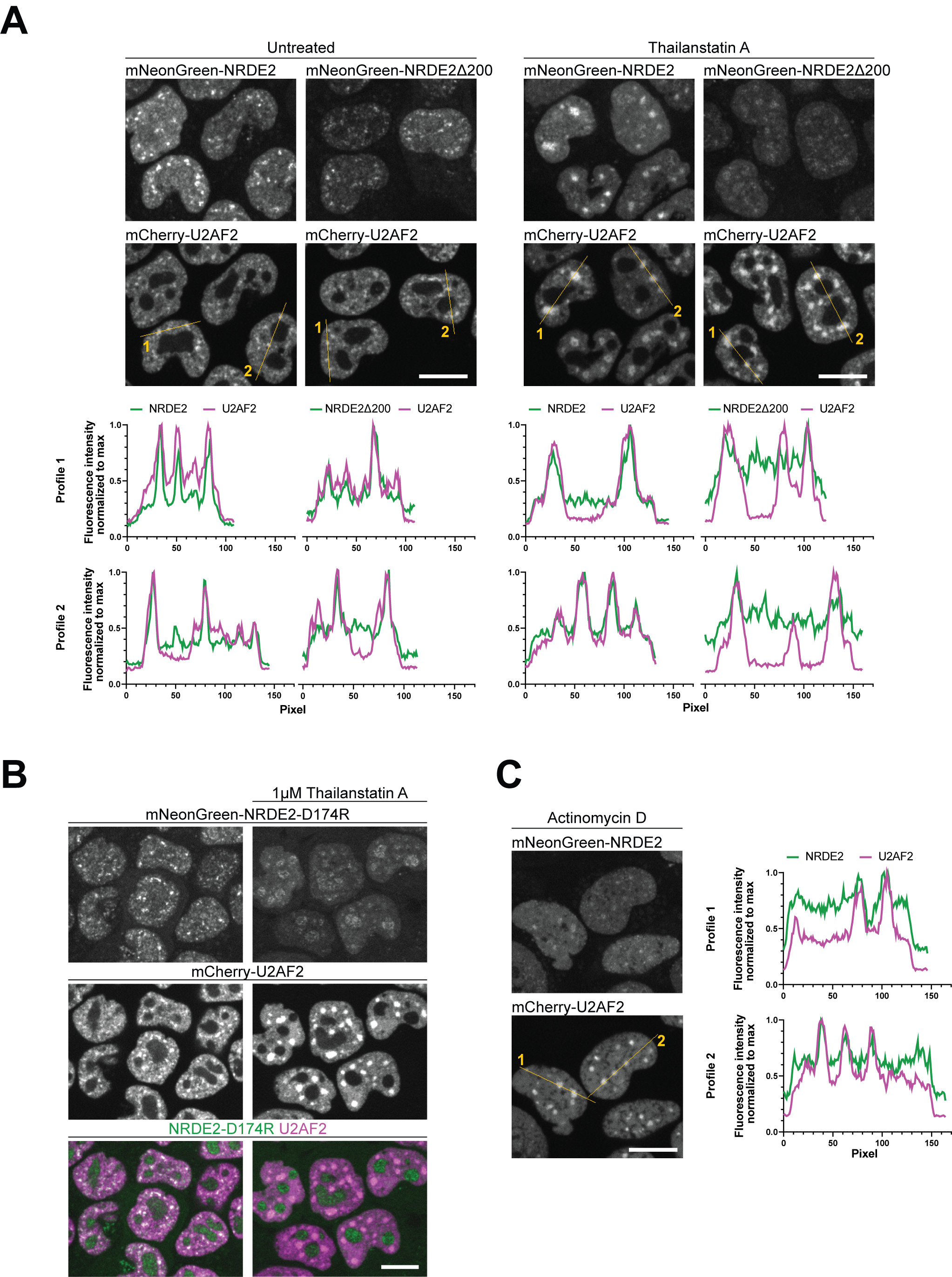

Supplement: Supplemental Material [file supp_079465.122_Supplemental_FigS3.tif]

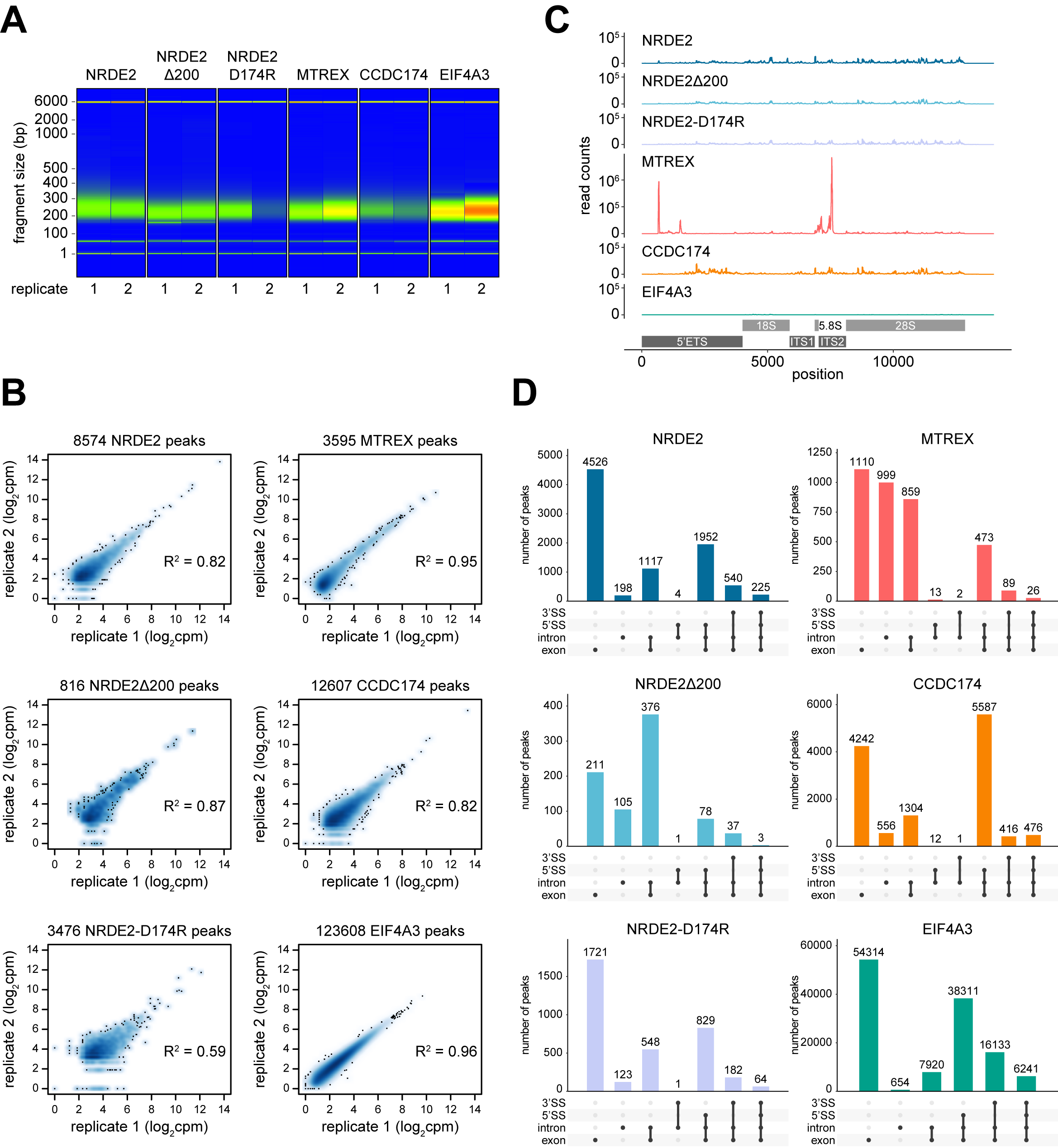

Supplement: Supplemental Material [file supp_079465.122_Supplemental_FigS4.tif]

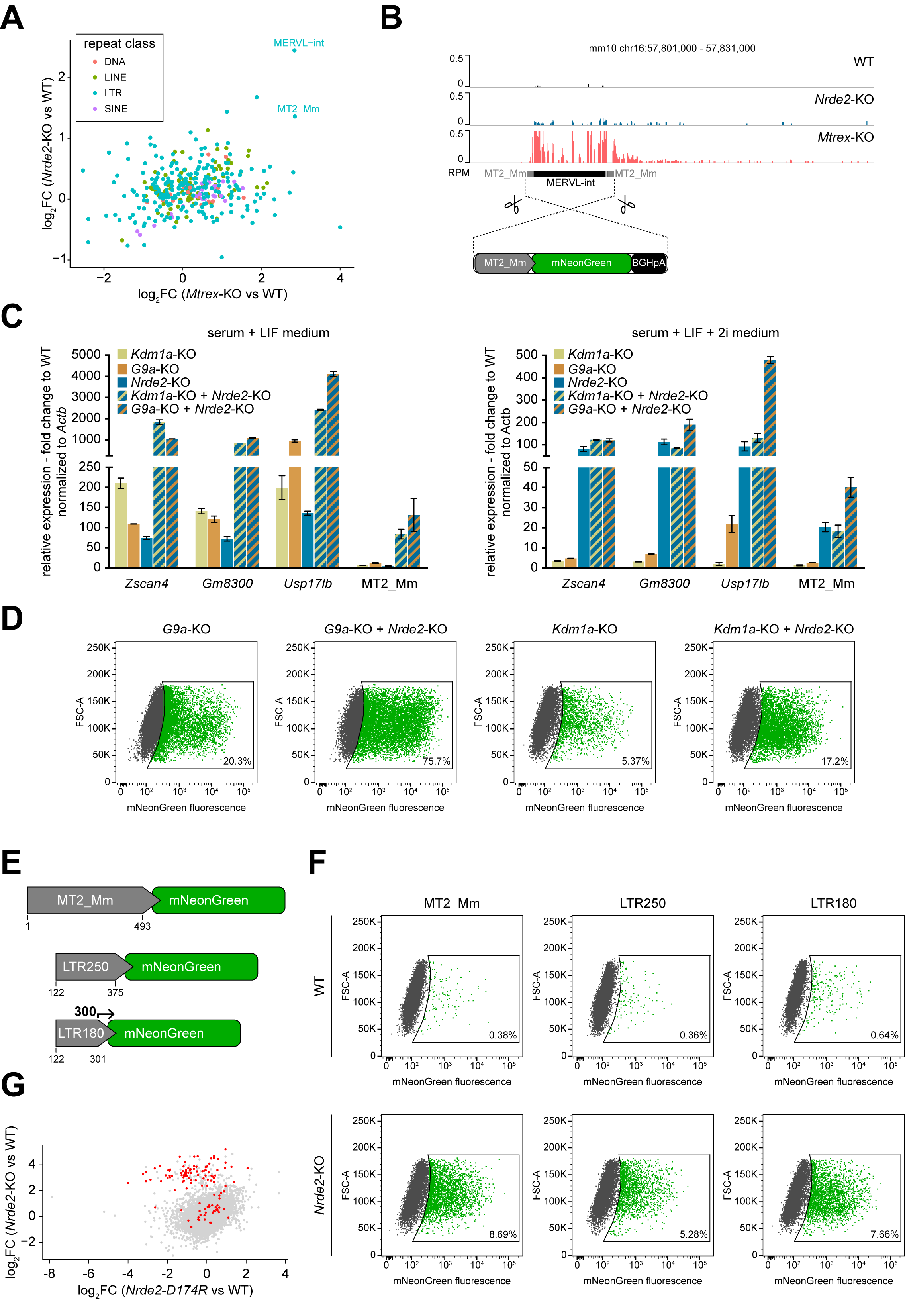

Supplement: Supplemental Material [file supp_079465.122_Supplemental_FigS5.tif]

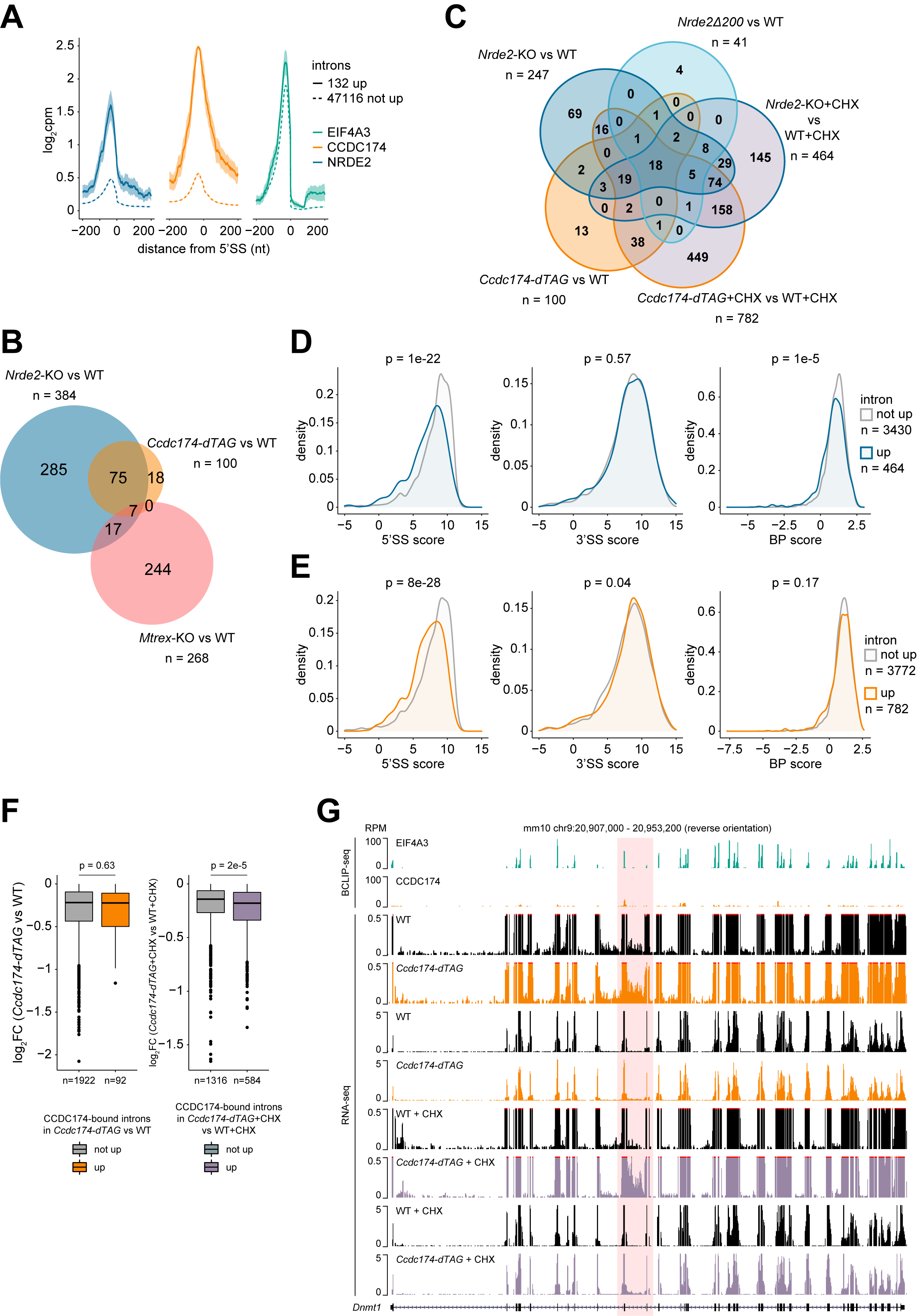

Supplement: Supplemental Material [file supp_079465.122_Supplemental_FigS6.tif]

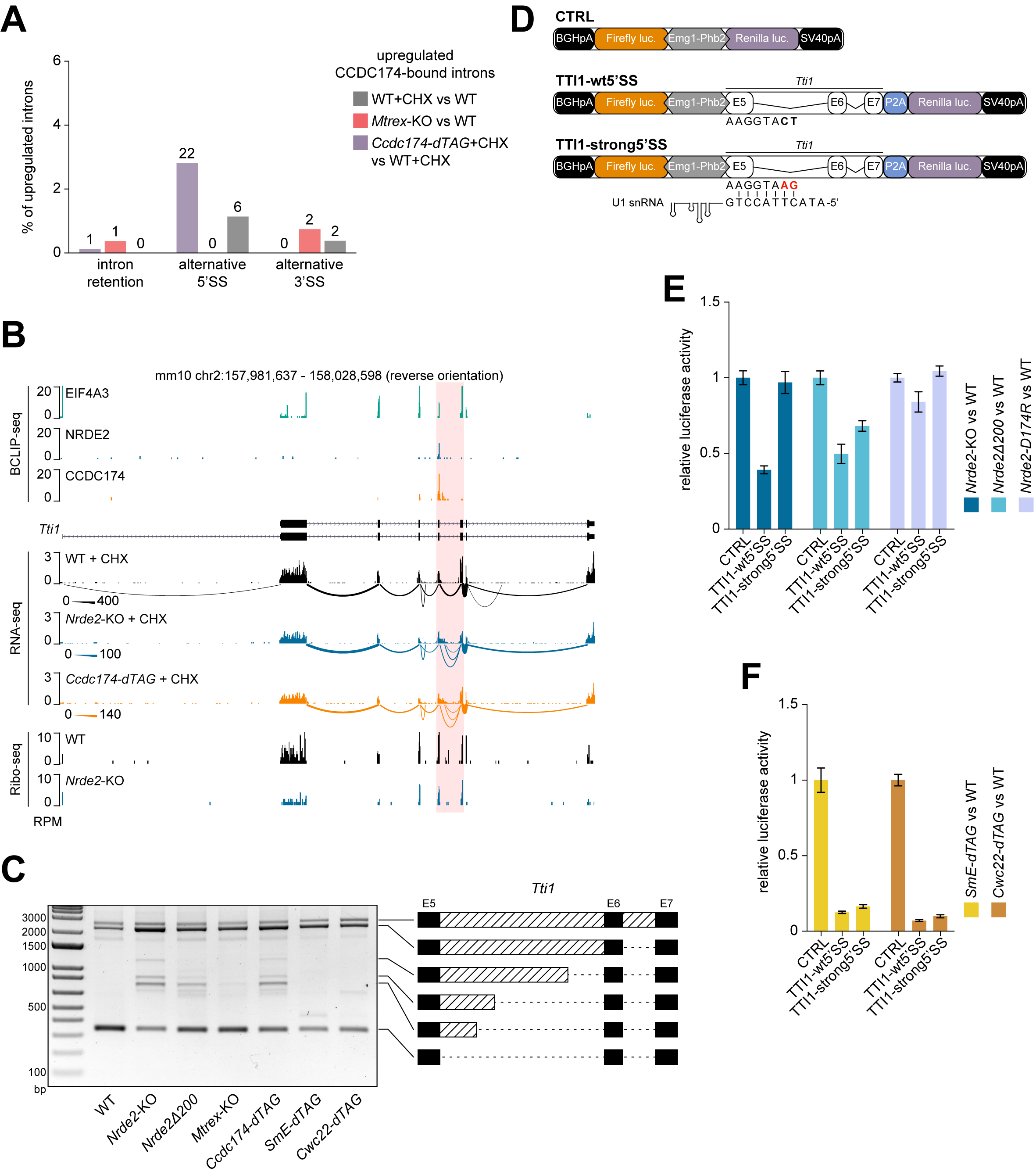

Supplement: Supplemental Material [file supp_079465.122_Supplemental_FigS7.tif]

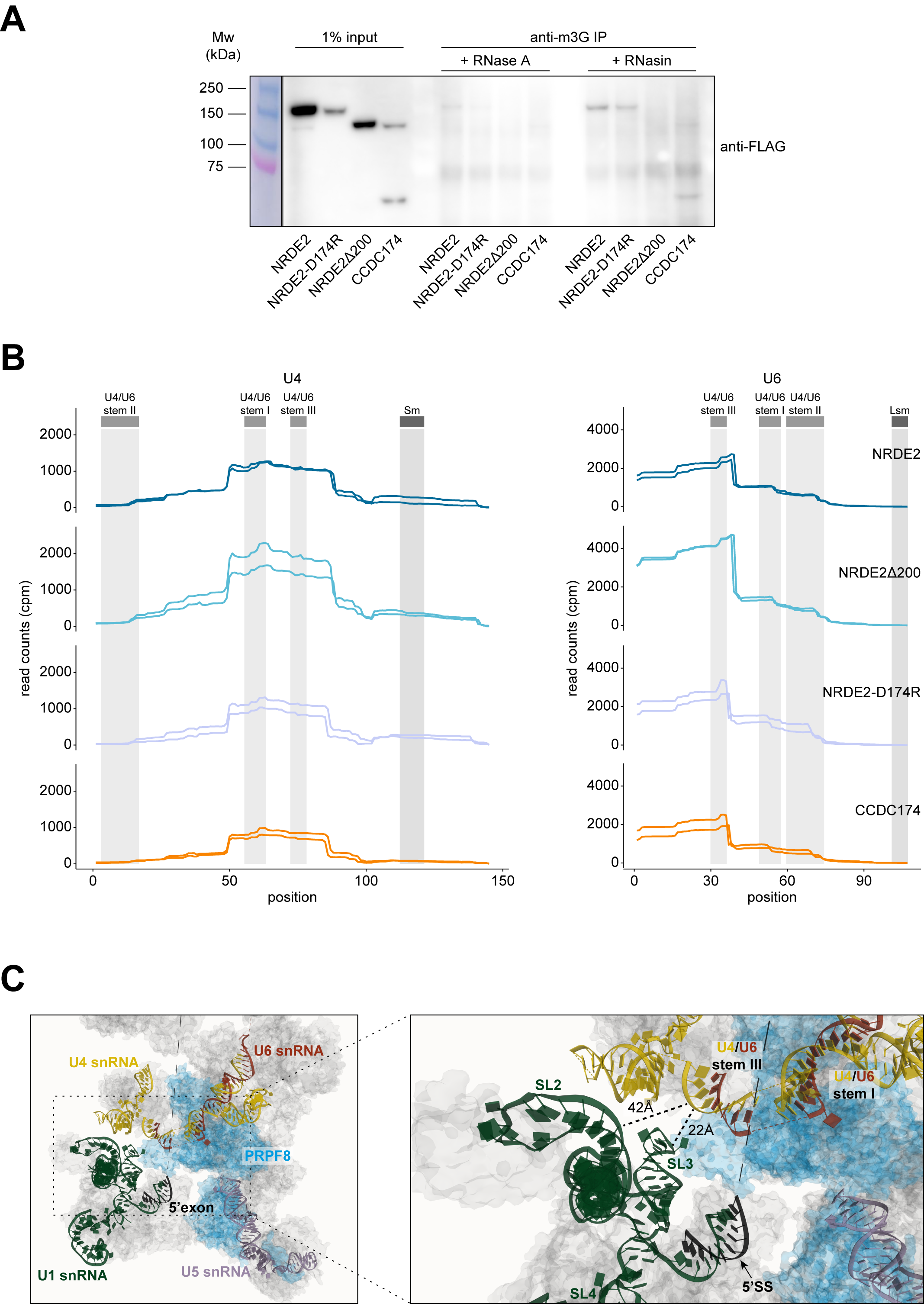

Supplement: Supplemental Material [file supp_079465.122_Supplemental_FigS8.tif]
