# Supplementary material for: Mouse nuclear RNAi-defective 2 promotes splicing of weak 5′ splice sites
Source: RNA. 2023 Aug;29(8):1140–65. doi: 10.1261/rna.079465.122 (PMC10351895; doi:10.1261/rna.079465.122)
Supplement: Supplemental Material [file supp_079465.122_Supplemental_Legends.docx]

**Figure S1. Overview of generated endogenously-tagged cell lines.**

(A) A schematic overview of TALEN- or Cas9-mediated genome editing manipulations performed in this study (See also Table S1).

(B) Heatmap of transcriptome correlations of parental untagged cells and all 3A-tagged and dTAG-tagged cells used in this study based on RNA-seq experiment performed in triplicates. cKO = uninduced conditional knockout.

(C) Time course of endogenous 3A-tagged NRDE2 levels upon conditional knockout induced by 0.1 μM 4OHT treatment, measured by Western Blotting with Streptavidin detection. The endogenous biotinylated proteins (marked by asterisks) serve as a loading control.

(D) Time course of endogenous CCDC174-dTAG depletion upon treatment with 0.5 μM dTAG-13, measured by Western Blotting with anti-HA antibody. The non-specific bands (marked by asterisks) serve as a loading control.

**Figure S2. Direct interaction with the MTREX helicase promotes NRDE2 stability.**

(A) Growth curve of 4OHT-inducible knockout *Nrde2*, *Nrde2Δ200* and *Nrde2-D174R* cells grown in the presence or absence of 0.1 μM 4OHT.

(B) Streptavidin pulldown and co-immunoprecipitation of endogenous 3A-MTREX from cells transiently overexpressing V5-tagged full length or truncated NRDE2 constructs fused to 2A-Puro. The detection of MTREX by FLAG antibody in NRDE2Δ100 lane was occluded on the reprobed membrane after the initial detection of V5-NRDE2Δ100 protein of identical size.

(C) Amino acid sequence alignment of MTREX-interacting region within mouse NRDE2 with its homologs from other vertebrates.

(D) Streptavidin pulldown and co-immunoprecipitation of endogenous 3A-MTREX from cells transiently overexpressing V5-tagged wild type or mutant NRDE2 constructs fused to 2A-Puro.

(E) Western blotting analysis of endogenous 3A-tagged NRDE2, NRDE2Δ200 and NRDE2-D174R protein expression. The strong band detected in all samples by streptavidin represents an endogenously biotinylated protein.

(F) Western blotting analysis of endogenous 3A-tagged NRDE2 expression in conditional *Mtrex*-KO cells untreated (WT) or treated for 3 days with 0.1 μM 4-OHT (KO). The upper band detected by anti-FLAG antibody represents a non-specific target as it is also present in the parental untagged cells.

(G) Native TAP-MS analysis of 3A-NRDE2-D174R under low salt conditions (100mM NaCl) compared to untagged control and performed in three independent replicates for each sample. Same labeling order as in Figures 1A and 1B applies.

(H) Formaldehyde cross-linking TAP-MS analysis of 3A-NRDE2-D174R compared to untagged control and performed in three independent replicates for each sample. Same labeling order as in Figures 1A and 1B applies.

**Figure S3. NRDE2 localization in nuclear speckles.**

(A) Quantification of endogenous mNeonGreen-tagged NRDE2 and NRDE2Δ200 colocalization with endogenous mCherry-tagged U2AF2 in untreated cells and cells treated for 6 hours with 1 μM splicing inhibitor Thailanstatin A (same conditions as in Figures 1G and 1H). Fluorescence signal intensity was quantified over two arbitrarily chosen lines depicted in yellow and plotted as normalized values with maximum intensity set to one. White line represents a scale bar of 15 μm.

(B) Live cell spinning disk microscopy of endogenous mNeonGreen-tagged NRDE2-D174R with endogenous mCherry-tagged U2AF2. The imaging was performed in untreated cells and cells treated for 6 hours with 1 μM Thailanstatin A. Scale bar 10 μm.

(C) Quantification of endogenous mNeonGreen-tagged NRDE2 colocalization with endogenous mCherry-tagged U2AF2 in cells treated for 2 hours with 5 μg/ml transcription inhibitor Actinomycin D (same conditions as in Figure 1I). Fluorescence signal intensity was quantified as in panel (A). White line represents a scale bar of 15 μm.

**Figure S4. BCLIP-seq allows reproducible detection of low-abundant protein-RNA interactions.**

(A) Gel image from Fragment Analyzer System (Agilent) displaying fragment size distribution of purified BCLIP-seq libraries. The combined size of adaptor sequences is 153 bp.

(B) Scatter plots showing the correlation of BCLIP-seq signal between two replicates of each assayed protein. The plotted signal represents normalized read counts in individual peaks identified by a peak calling analysis.

(C) BCLIP-seq signal coverage over the first 14 kb of mouse ribosomal DNA repeat unit (GenBank: BK000964). Positions of annotated rRNA segments are provided, ETS = external transcribed spacer, ITS = internal transcribed spacer.

(D) UpSet plots displaying the overlap of called BCLIP-seq peaks with different pre-mRNA features and their intersections. Each bar shows a number of peaks overlapping the features marked by a black dot. The features were extracted from the GENCODE transcriptome annotation which may include multiple alternatively spliced transcript isoforms for a single gene. For example, the category exon+intron (third bar) comprises peaks overlapping an alternative exon that is annotated as an exon in some transcript isoforms and as an intron in other isoforms of a given gene.

**Figure S5. NRDE2 depletion leads to MERVL and 2C gene activation.**

(A) Differential expression of repetitive elements in *Nrde2*-KO and *Mtrex*-KO cells relative to the corresponding WT cells. Each dot represents a unique repeat type based on RepeatMasker annotation.

(B) UCSC genome browser screenshot showing normalized RNA-seq tracks at a full length MERVL-containing locus on chromosome 16. Below is a schematic overview of the 2C reporter and its genomic insertion strategy.

(C) Quantitative RT-PCR analysis of MT2_Mm and selected 2C marker genes expression in *Kdm1a*-KO, *G9a*-KO, *Nrde2*-KO cells and their combinations relative to WT cells grown in the presence or absence of 2i inhibitors. Plotted is the average of two independent experiments performed in technical triplicates and the error bars represent standard deviation.

(D) Flow cytometry analysis of stably integrated 2C reporter expression in *G9a*-KO and *Kdm1a*-KO cells with or without simultaneous induced knockout of *Nrde2* (6 days of 0.1 μM 4OHT treatment). Cells were grown in serum + LIF medium in the absence of 2i inhibitors.

(E) Schematic depiction of the 2C reporter variants driven by MT2_Mm fragments of different length. The bent arrow indicates the transcriptional start site determined by 5'RACE.

(F) Flow cytometry analysis of the expression of stably integrated 2C reporter variants described in panel (E) in WT and *Nrde2*-KO cells (6 days of 0.1 μM 4OHT treatment).

(G) Scatter plot comparing differential gene expression in *Nrde2*-KO and *Nrde2-D174R* cells relative to WT cells. Each dot represents a single gene. Genes upregulated in 2C-like cells (Macfarlan et al., 2012) are highlighted in red.

**Figure S6. CCDC174 regulates splicing of introns with weak 5'splice sites.**

(A) Metaplots of normalized NRDE2, CCDC174 and EIF4A3 BCLIP-seq signal around 5'SSs of introns upregulated (solid line) or not upregulated (dashed line) in *Ccdc174-dTAG* cells compared to WT.

(B) Overlap of CCDC174 target introns (introns with a CCDC174 BCLIP-seq peak at their 5'SS) upregulated in *Nrde2*-KO, *Ccdc174-dTAG* or *Mtrex*-KO cells.

(C) Overlap of NRDE2 target introns upregulated in *Nrde2*-KO cells untreated or treated for 4 hours with 100 μg/ml cycloheximide (CHX) and in *Nrde2Δ200* cells with CCDC174 target introns upregulated in *Ccdc174-dTAG* cells untreated or treated with CHX relative to their respective CHX-treated or untreated WT controls.

(D) Density plots of the distribution of NRDE2 target introns upregulated (blue line) or not upregulated (grey line) in *Nrde2*-KO cells treated with CHX based on their 5'SS, 3'SS and branch point strength. P-values were calculated using Wilcoxon rank-sum test.

(E) Density plots of the distribution of CCDC174 target introns upregulated (orange line) or not upregulated (grey line) in *Ccdc174-dTAG* cells treated with CHX based on their 5'SS, 3'SS and branch point strength. P-values were calculated using Wilcoxon rank-sum test.

(F) Differential expression of genes containing CCDC174 target introns in *Ccdc174-dTAG* cells (24 hours of 0.5 μM dTAG-13 treatment) untreated (left) or treated (right) with CHX. Genes are split into two groups: those containing only target introns not upregulated (grey) and those containing at least one target intron upregulated in *Ccdc174-dTAG* cells untreated (orange) or treated with CHX (purple). P-values were calculated using Wilcoxon rank-sum test.

(G) UCSC genome browser screenshot showing normalized BCLIP-seq and RNA-seq tracks over *Dnmt1* gene locus. Position of the CCDC174 target intron 10 is highlighted in red.

**Figure S7. NRDE2 regulates splicing of its target gene *Tti1*.**

(A) rMATS analysis of alternative splicing events within CCDC174 target introns upregulated (inclusion level difference > 0.1) in *Mtrex*-KO cells and in CHX-treated WT and *Ccdc174-dTAG* cells relative to their corresponding WT and untreated controls (only introns with > 30 reads across all samples were included in the analysis).

(B) UCSC genome browser screenshot showing normalized BCLIP-seq, RNA-seq and Ribo-seq tracks over *Tti1* gene locus combined with a Sashimi plot of *Tti1* splicing patterns detected in the RNA-seq data. The thickness of the splice junction connecting lines reflects the number of corresponding spliced RNA-seq reads with a scale provided on the left. NRDE2 and CCDC174 target intron 5 is highlighted in red.

(C) RT-PCR analysis of *Tti1* intron 5 splicing with primers binding in exon 5 and 7. The additional bands in *Nrde2*-KO sample were sequenced and the identified cryptic 5'SSs are schematically depicted.

(D) Schematic overview of the TTI1 dual luciferase reporters. The mutations introduced in *Tti1* intron 5 5'SS to improve U1 snRNA complementarity in the TTI1-strong5'SS reporter are depicted in red.

(E and F) Dual luciferase assay of the transiently transfected TTI1 reporters. Plotted is the average value of normalized Renilla luciferase activity from three independent experiments each performed in triplicate transfections. Error bars represent standard deviation.

**Figure S8. NRDE2 and CCDC174 contacts with snRNAs fit the pre-catalytic spliceosome model.**

(A) Anti-m3G cap pulldown and co-immunoprecipitation of endogenously 3A tagged NRDE2, NRDE2-D174R, NRDE2Δ200 and CCDC174 in the presence of RNase A or the RNase inhibitor RNasin.

(B) BCLIP-seq signal coverage over U4 and U6 snRNA sequences showing the two BCLIP-seq replicates of each sample separately. Grey columns indicate the positions of the Sm/LSm ring binding sites and the three U4/U6 base-pairing regions.

(C) Snapshot of the human spliceosomal pre-B complex structure PDB ID: 6QX9 (Charenton et al., 2019) showing the proximity of U1 snRNA stem-loops II and III with U4/U6 snRNA stem III. NRDE2 and CCDC174 xTAP-MS interactor PRPF8 is depicted in blue. The black dotted lines in the close-up image on the right depict the closest distance between U1 stem-loops II and III and U4/U6 stem III with the measurement value included.

**Table S1.** A list of mES cell lines generated and used in this study, including the details of all genome editing steps.

**Table S2.** A complete list of NRDE2 interactors identified in a yeast two-hybrid screen of full length *Nrde2* cDNA against an mES cell library.

**Table S3.** Description of oligos used for BCLIP-seq library construction.

**Table S4.** A list of primers used for qPCR and for splicing pattern PCR analysis.
